# Evolution of mammalian longevity: age-related increase in autophagy in bats compared to other mammals

**DOI:** 10.18632/aging.202852

**Published:** 2021-03-21

**Authors:** Joanna Kacprzyk, Andrea G. Locatelli, Graham M. Hughes, Zixia Huang, Michael Clarke, Vera Gorbunova, Carlotta Sacchi, Gavin S. Stewart, Emma C. Teeling

**Affiliations:** 1 School of Biology and Environmental Science, University College Dublin, Belfield, Dublin 4, Ireland; 2 Departments of Biology and Medicine, University of Rochester, Rochester, NY 14627, USA; 3 Present Institutional Address: Division of Genetics and Cell Biology, Fondazione Centro San Raffaele, Via Olgettina, Milano 6020132, Italy

**Keywords:** autophagy, bats, aging, blood mRNA, phylogenomics

## Abstract

Autophagy maintains cellular homeostasis and its dysfunction has been implicated in aging. Bats are the longest-lived mammals for their size, but the molecular mechanisms underlying their extended healthspan are not well understood. Here, drawing on >8 years of mark-recapture field studies, we report the first longitudinal analysis of autophagy regulation in bats. Mining of published population level aging blood transcriptomes (*M. myotis*, mouse and human) highlighted a unique increase of autophagy related transcripts with age in bats, but not in other mammals. This bat-specific increase in autophagy transcripts was recapitulated by the western blot determination of the autophagy marker, LC3II/I ratio, in skin primary fibroblasts (*Myotis myotis,* *Pipistrellus kuhlii*, mouse), that also showed an increase with age in both bat species. Further phylogenomic selection pressure analyses across eutherian mammals (n=70 taxa; 274 genes) uncovered 10 autophagy-associated genes under selective pressure in bat lineages. These molecular adaptations potentially mediate the exceptional age-related increase of autophagy signalling in bats, which may contribute to their longer healthspans.

## INTRODUCTION

Understanding the aging process with a view of managing/reducing its ailments is crucial to improve the quality of life of our aging populations [1]. The hallmarks of aging are remarkably similar across mammals, but the rate vastly differs [2] and the molecular basis for this natural variation in longevity is not well understood. This suggests that studying the aging process in exceptionally long-lived species, such as bats, will enable us to elucidate the mechanisms underlying naturally evolved longer healthspans and ultimately contribute to a greater understanding of aging biology [3]. Relative to body mass, bats show the longest lifespans of all mammals and exhibit little signs of senescence [4, 5]. For this reason, bats are now being recognised as novel, relevant models to study the mechanisms of healthy aging. Comparative studies focused on bats have furthered our understanding of variation in aging across the mammal tree of life and suggested factors that may underlie their extended healthspans: telomeres [5], mitochondria [6], microbiome [7] and metabolome [8]. A recently published longitudinal study highlighted that bats exhibit a unique, age-related gene expression pattern associated with DNA repair, immunity and autophagy [9]. Indeed, autophagy and proteostasis were previously suggested to be the common mechanisms that maintain health in long-lived species, including bats [10–12]. Enhanced autophagy has also been suggested as an anti-viral mechanism in *Rousettus* bats [13] which may also contribute to bat’s unique longer healthspans [14]. However until now, studying the age-dependent changes of autophagy in wild bat populations has been hindered by the logistical challenges [5].

Autophagy is a convergent mechanism of multiple longevity pathways, playing a role in lifespan extension promoted by reduced insulin/IGF-1, mTOR inhibition and dietary restriction in mammals [15]. Functional studies in model species demonstrate that reduced autophagy shortens lifespan, while increased autophagy extends it [16]. Accordingly, many studies have demonstrated that autophagy decreases with age, and it has been inferred that this gradual decrease could play a major role in the functional deterioration of aging organisms [17].

To ascertain if autophagy is involved in bats’ exceptional longevity, we firstly mined our published longitudinal bat blood transcriptomes [9] and identified 26 autophagy-associated genes that are up-regulated with age in long-lived wild *M. myotis* (Greater mouse-eared bat), but down-regulated in human and mouse. Secondly, drawing on two long-term mark-recapture studies of wild populations of *Myotis myotis* and *Pipistrellus kuhlii* (Kuhl’s pipistrelle), we sampled wing-biopsies from bats across known ages, generated primary fibroblast cell lines, and demonstrated the increase of the autophagy marker, LC3II/I ratio, with age in both bat species, but not in mice. In order to identify the genomic adaptations underlying this unique age-related increase of autophagy signalling in bats, we carried out a eutherian-wide (n=70 species, including both *M. myotis* and *P. kuhlii* bats) comparative phylogenomic analyses of 274 autophagy-associated genes. Despite the high conservation of autophagy pathways, 10 genes showed unique evolutionary signatures of selection in bat lineages. These integrative data provide a multi-layered insight into bats’ autophagy signalling and suggest that molecular adaptation of autophagy pathways may underly bats longer healthspans.

## RESULTS

### Bat-specific changes in expression of autophagy-associated genes with age

To explore the age-related changes in expression of autophagy associated genes we mined the aging blood transcriptomes from *M. myotis*, human and mouse that were previously generated in our lab [9]. The Spearman’s rank correlation coefficients were extracted for 70 autophagy associated genes present in the dataset and pathway analysis suggested that autophagy GO terms show different patterns during aging in bats, compared to humans and mice (Table 1A). In particular, 26 genes showed increasing expression with age in *M. myotis* bats, while they were downregulated in both humans and mice (Table 1B).

**Table 1A:** Comparative transcriptomic analyses between bat, human, mouse.

			**Bat**	**Human**	**Mouse**
**GO:0006914**	autophagy	62	0.194	-0.095	-0.003
**GO:0016236**	macroautophagy	47	0.197	-0.116	-0.026
**GO:0010508**	positive regulation of autophagy	16	0.204	-0.05	-0.05
**GO:0010506**	regulation of autophagy	44	0.196	-0.065	-0.018
**GO:0016241**	regulation of macroautophagy	25	0.209	-0.083	-0.05

**Table 1B:** Comparative transcriptomic analyses between bat, human, mouse.

**Gene**	**Bat**	**Human**	**Mouse**
*PIK3CA*	0.483	-0.233	-0.568
*ROCK1*	0.468	-0.091	-0.41
*RB1CC1*	0.446	-0.143	-0.164
*CSNK2A1*	0.415	-0.015	-0.406
*NEDD4*	0.383	-0.043	-0.1
*PAFAH1B2*	0.293	-0.278	-0.097
*USP33*	0.278	-0.237	-0.165
*TRAPPC8*	0.274	-0.167	-0.066
*ULK2*	0.271	-0.017	-0.312
*SIRT1*	0.243	-0.091	-0.566
*SNX14*	0.214	-0.27	-0.158
*VTA1*	0.208	-0.302	-0.327
*MAP3K7*	0.203	-0.213	-0.247
*TBK1*	0.198	-0.218	-0.215
*VPS36*	0.179	-0.207	-0.483
*NRBF2*	0.177	-0.236	-0.361
*DNM1L*	0.168	-0.081	-0.245
*UBXN2B*	0.146	-0.14	-0.179
*EIF2AK4*	0.141	-0.069	-0.361
*SH3GLB1*	0.132	-0.149	-0.039
*PIK3R4*	0.11	-0.227	-0.457
*ATP6V1H*	0.099	-0.172	-0.032
*PIK3C3*	0.077	-0.268	-0.269
*RAB3GAP1*	0.053	-0.013	-0.567
*ATP6V1C1*	0.03	-0.186	-0.09
*U2AF1*	0.03	-0.053	-0.207

(A) Comparison of the pathway expression pattern for autophagy-associated GO terms. Within each species, the median of the Spearman’s correlation coefficients of all genes under each of enriched age-associated GO terms was used to represent their overall expression pattern with age. The values behind the GO terms indicate the number of genes enriched. (B) The autophagy associated genes that exhibited the opposite direction of expression changes with age in bat compared to human and mouse are shown. Values indicate the Spearman’s correlation coefficients between gene expression and age for each species.

### LC3II/I ratio increases with age in bat skin-derived fibroblasts

The age-associated changes in LC3II/I ratio under control and serum withdrawal conditions were investigated using western blot in primary fibroblasts derived from female individuals. The experiments were limited to females since the bat captures were carried out in the maternity roosts and as a result the sampled individuals were predominantly female. The two bat species included in this study live longer than expected given their body mass. The calculated longevity quotient (LQ=observed/expected longevity) was 1.6 for *P. kuhlii* and 5.7 for *M. myotis*, in contrast to 0.6 for mouse, included as a control system. Wing skin biopsies (bats) or small ear skin (mouse) clippings were successfully used as a source of primary fibroblasts, with between 200 to 600k cells obtained per sample after 9-10 days of culture. In the pilot experiments, attempts to expand the fibroblast cultures beyond this point often resulted in reduced growth rate, therefore cells were not passaged further. Generally, samples from 6+ and 7+ years old *P. kuhlii* and 22-month old mouse grew at similar rate to the rest of the age ranges, but a smaller number of initial fibroblasts growth halos were produced, resulting in the lower final cell numbers available for experiments.

**Figure 1:**
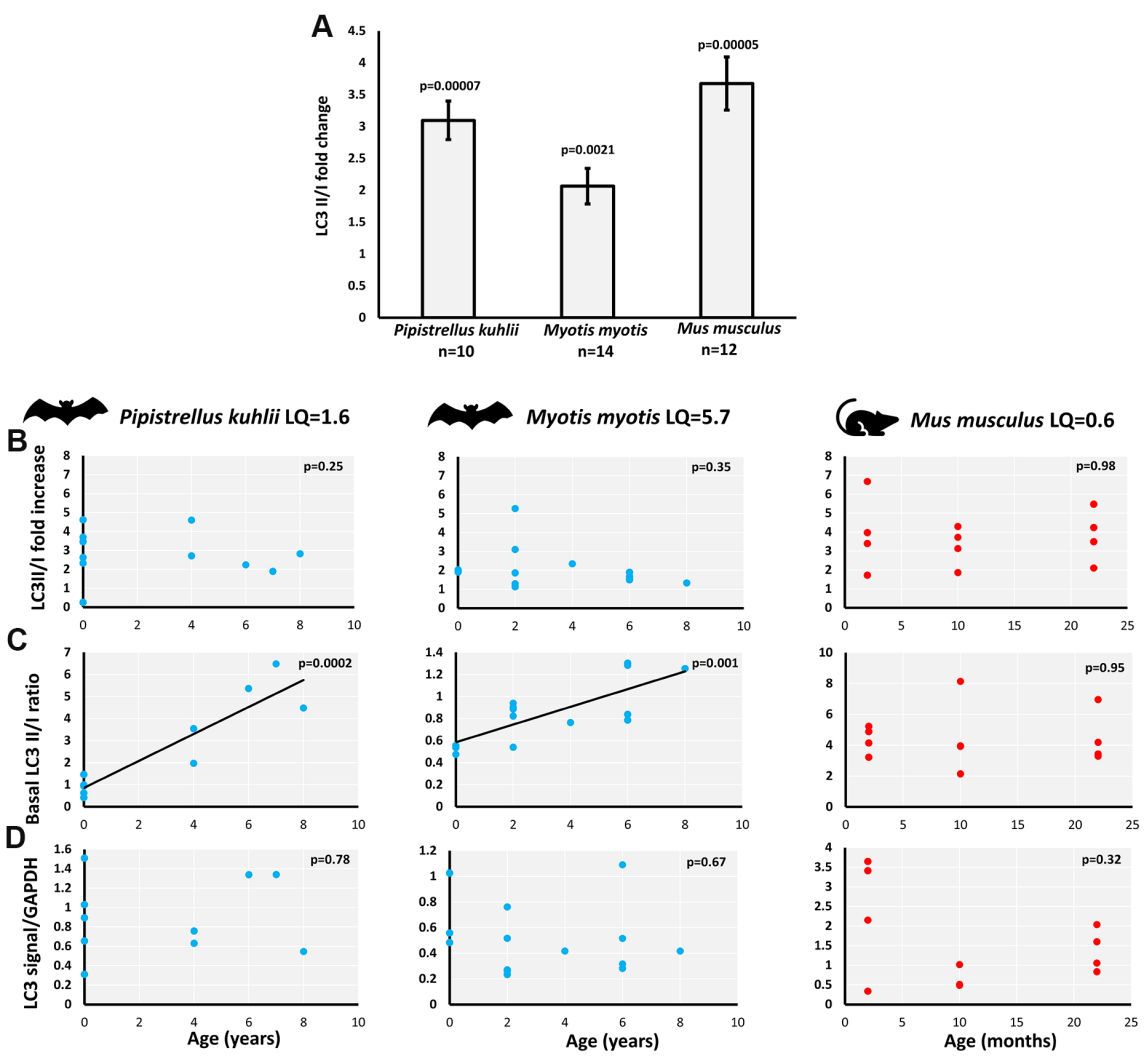
**Basal and starvation-induced autophagy in skin-derived fibroblasts from *P. kuhlii*, *M. myotis* and *M. musculus*.** (**A**) fold change of LC3 II/I ratio induced by serum withdrawal (p-values: two-tailed t-test; data represent mean ±SEM). Relationship between individual’s age and (**B**) starvation-induced LC3II/I fold change, (**C**) basal LC3 II/I ratio, (**D**) GAPDH normalized total LC3 signal. (**B**–**D**) Corresponding *p*-values indicate the significance of linear model and are indicated in the top right-hand corner of each plot. Models are plotted where significant. LQ – longevity quotient. Note that scales differ between species.

LC3II/I ratio measures conversion of the microtubule-associated protein 1 light chain 3 (LC3), from a free LC3I form to lipidated LC3II form associated with autophagosomes. As anticipated, skin-derived fibroblasts of all species (*P. kuhlii*, *M. myotis* and *M. musculus*) responded to serum withdrawal treatment with increase of LC3II/I ratio (Figure 1A). The increase of LC3II/I ratio in *M. myotis* and *P. kuhlii* fibroblasts induced by pharmacological treatment with rapamycin, another classic inducer of autophagy [18] additionally validated the use of this autophagy marker in bats (Supplementary Figure 1).

We avoided direct cross-species comparisons and focused on age-related changes within each species, as there might be species-specific differences in antibody affinity for LC3-I compared to LC3-II. The starvation-induced LC3II/I fold-change was not correlated with age in either bat species nor in mouse (Figure 1B). However, the basal LC3II/I ratio significantly increased with age in *P. kuhlii* and *M. myotis*, but not in mouse (Figure 1C). Lack of LC3II/I increase with age in mice did not change after including samples from different genetic backgrounds, nor when derived from flank skin rather than ear skin [19] (Supplementary Figure 2). Total LC3 signal (LC3II + LC3I normalized to GAPDH) did not show age-related changes in any of the tested species (Figure 1D).

**Figure 2:**
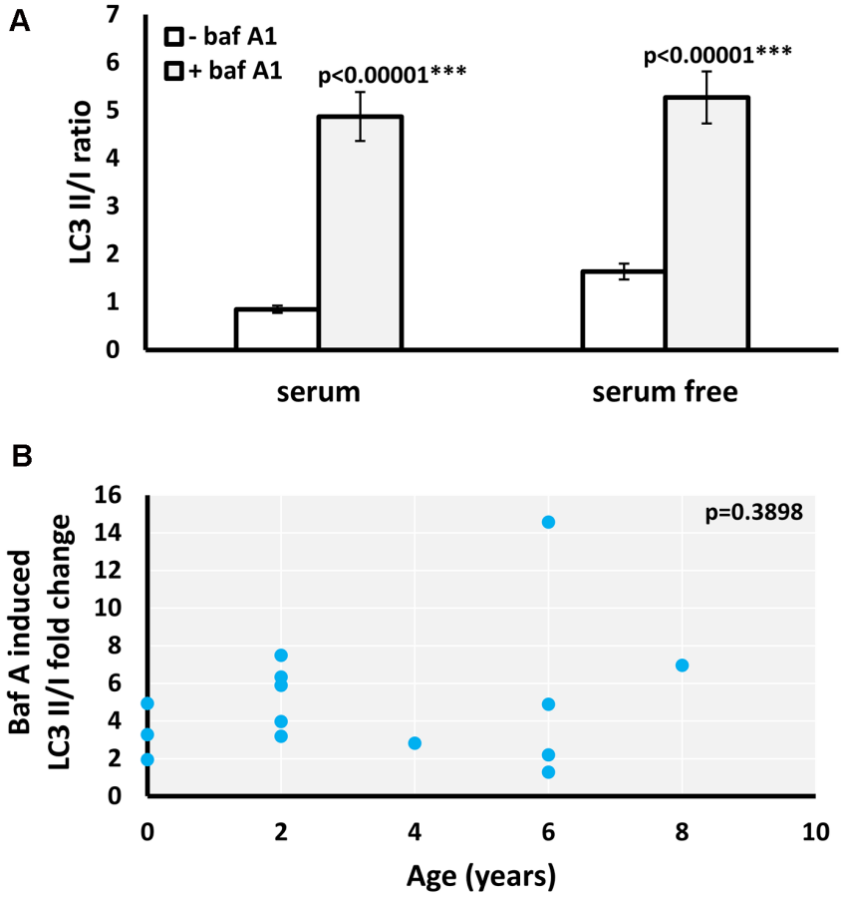
**Effect of baf A1 autophagy block on basal and serum withdrawal-induced autophagic flux in *M. myotis* fibroblasts.** (**A**) Basal and starvation-induced LC3II/I ratio in absence and presence of 100nM baf A1, data represent means ±SEM for n=14 individuals age 0 to 8. p-values (two-tailed t-test) indicate statistically significant effect of baf A1 treatment. (**B**) Relationship between individual’s age and baf A1 induced fold change of basal (serum present) LC3II/I ratio (p-value included in the top right-hand corner of the plot indicates that linear model is not significant).

Increased LC3II/I ratio can be a result of either upregulation of autophagosome formation or a blockage of autophagic degradation [20]. To determine if the age-related increase of LC3II/I ratio observed results from defective autophagic degradation, we used bafilomycin A1 (Baf A1), an inhibitor of fusion between autophagosomes and lysosomes [21]. Both the basal and starvation-induced LC3II/I ratio significantly increased in the presence of Baf A1 in *M. myotis* (Figure 2A). Moreover, there was no significant age-associated change in the effect of Baf A1 on the basal LC3II/I ratio (Figure 2B), indicating that the age-related increase in basal LC3II/I ratio was likely not due to defective autophagy. Due to low numbers of cells obtained for the oldest cohort of *P. kuhlii* (6-8 years) it was not possible to include the autophagy inhibitor treatment for this species. Representative gels for all experiments are included in the Supplementary Figure 3.

### Assembly of *P. kuhlii* transcriptome

Given the lack of an assembled *P. kuhlii* genome, we isolated coding sequences (CDSs) of autophagy-associated genes suitable for selection tests, from our sequenced *P. kuhlii* fibroblast transcriptomes. To maximize the chance of capturing transcripts up- and down- regulated upon induction of autophagy, both control (n=3) and serum-starved (n=3) samples were sequenced (Supplementary Table 1A). *De novo* pooled transcriptome assembly yielded a total of 271,767 transcripts (Supplementary Table 1B), of which 109,942 were annotated as protein-coding, corresponding to 15,542 unique genes. Twenty-four percent of autophagy associated genes retrieved using search term ‘autophagy’ from AmiGO database [22], exhibited differential expression under serum starvation conditions (Supplementary Table 2).

### The signatures of positive and divergent selection in autophagy-associated genes in bats

**Figure 3:**
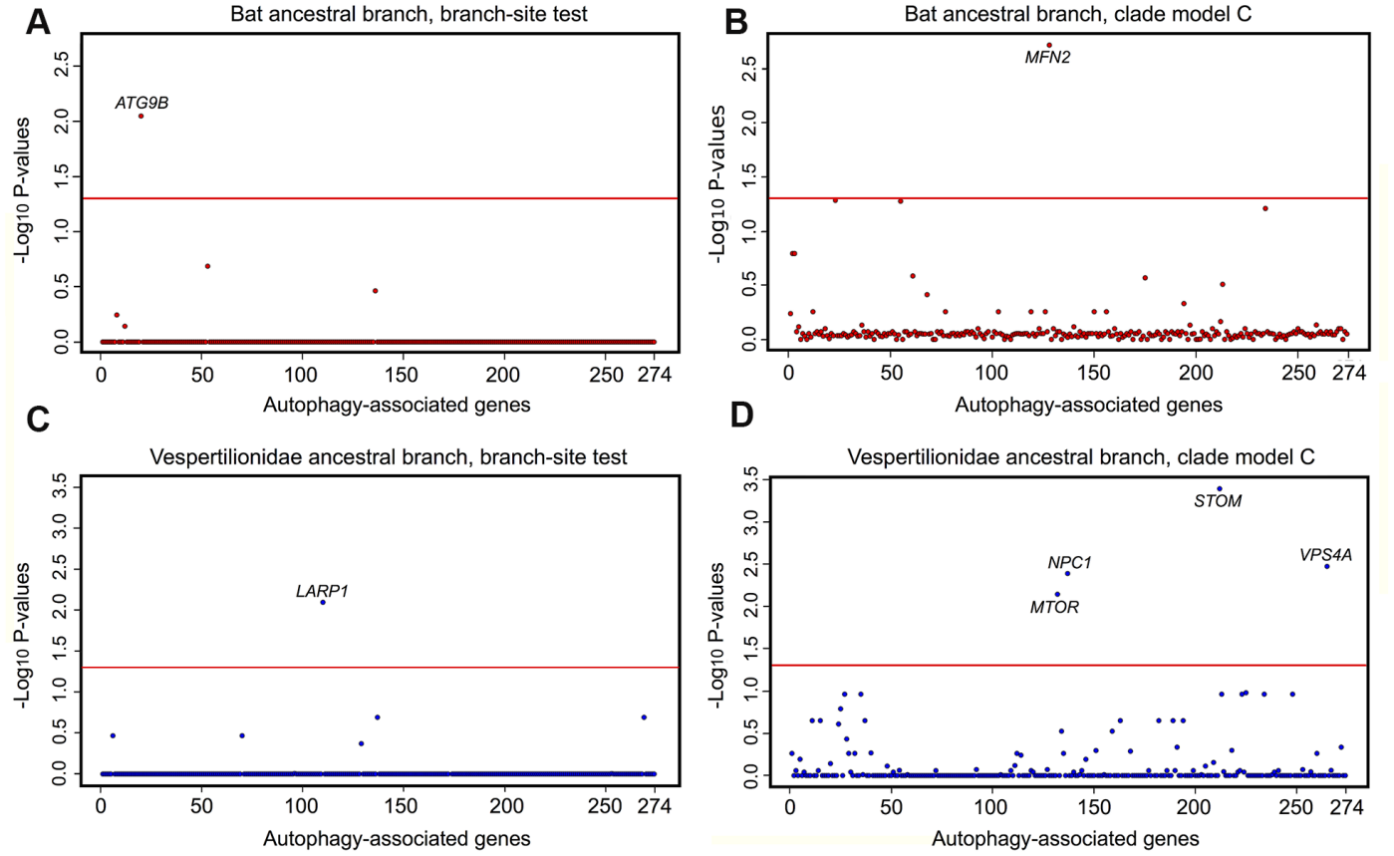
**Selective pressure acting on autophagy-associated genes (n=274) in bat lineages.** Results of tests for positive and divergent selection using the CodeML branch-site and clade model C models, conducted on the (**A**, **C**) the bat ancestor branch, (**B**, **D**) the vespertilionid ancestor. *P* values are transformed using −log_10_. Genes significant after FDR correction and appearing in both RefSeq and RefSeq+MAKER (including extra 8 species with highly fragmented genome assemblies) data sets are labelled above the red line, which indicates a significance cut-off of α = 0.05.

Phylogenomic selection tests were carried out on a suite of autophagy associated genes across eutherian mammals. Tested genes were: i) GO-associated with term autophagy; ii) represented by at least 50% of the 62 mined eutherian genomes; and, iii) detected in the assembled *P. kuhlii* transcriptome. Supplementary Figure 4 presents the outline of workflow used to isolate the final set of 274 genes for selection analyses. Tests of positive and divergent selection were carried out independently along the bat lineages and mouse (Supplementary Figure 5). CodeML calculates the likelihood-derived dN/dS rates (ω), where dN is defined as a number of non-synonymous substitutions per non-synonymous sites and dS is a number of synonymous substitutions per synonymous sites. Positive selection (ω >1) was detected in *ATG9B* along the ancestral bat branch and for *LARP1* along the ancestral vespertilionid branch, with both genes showing significant sites under selection (Figure 3A, 3B and Supplementary Table 4). A number of sites had significant BEB scores for *ATG9B* in the *Myotis* ancestral branch, however these were present only in *M. lucifugus*, and represented a missing exon in other *Myotis* taxa. Within the individual lineages, positive selection was found in VMP1 and ZDHHC8 for *P. kuhlii*. Divergent selection was detected in MFN2 (ancestral bat branch) and in *MTOR, STOM, VPS4A* and *NPC1* (ancestral vespertilionid branch) (Figure 3C, 3D and Supplementary Table 3). The ω values for *MFN2, MTOR, STOM, VPS4A* were >1 in the foreground and <1 in the background branch, indicating positive divergent selection acting along respective foreground branches. However, for *NPC1* both foreground and background ω fell within the region of purifying selection with values of 0.12 and 0.24 respectively, suggesting that this gene may be under extreme evolutionary constraint and thus essential for normal cellular function. In the individual lineages, divergent selection was found in *SFRP4* for the *P. kuhlii* (foreground ω>1, background ω<1). No genes found under selection in bats were found under selection in *M. musculus,* where positive selection was found in *PSAP* and divergent selection was found in *SNX14* (foreground ω<1, background ω>1) (Supplementary Table 3) showing different evolutionary pressures acting on autophagy pathways in bats and mice.

**Figure 4:**
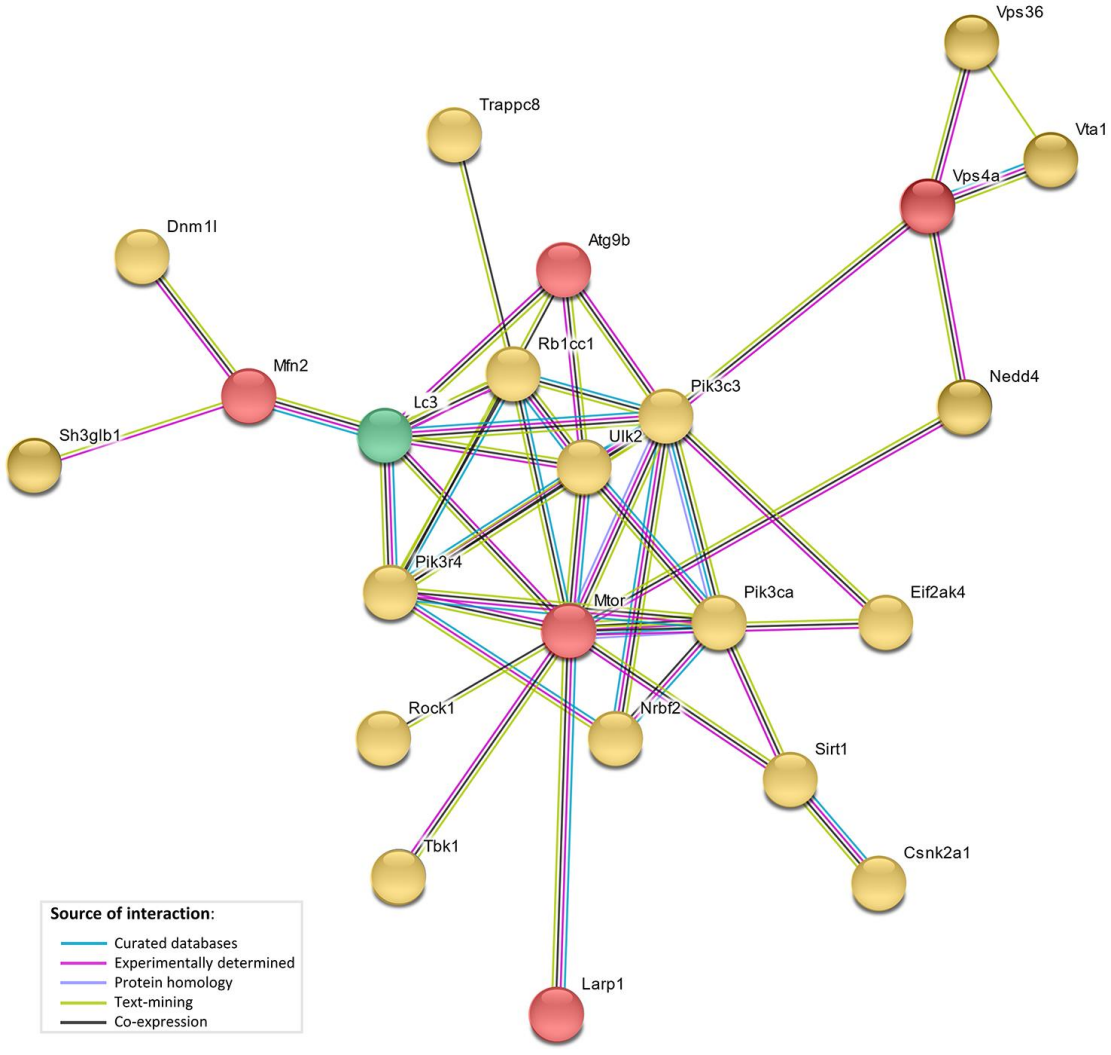
**STRING interaction network.** STRING protein-protein interaction network showing direct interactions between genes under selective pressure in bat lineages (red), genes with bat-specific upregulation with age (yellow), and LC3 (green).

The network analysis (STRING database v.10. [23]) showed direct interaction between 5 genes under selection in the bat lineages (*LARP1*, *MTOR*, *ATG9B*, *VPS4A* and *MFN2*), and with 17 of autophagy-associated genes which positively correlated with age, and with the LC3 protein (Figure 4). This network showed a significant functional enrichment (FDR corrected p-value <0.05) for 152 Biological Process GO terms and 14 KEGG (Kyoto Encyclopedia of Genes and Genomes) pathways (Supplementary Tables 4, 5), including terms/pathways involved in the regulation and early events of autophagic activity.

## DISCUSSION

The essential role of autophagy in lifespan regulation is supported by extensive evidence from studies on model species, including mouse, nematodes and flies, as well as humans [16, 24]. Autophagy is now one of the most studied phenomena in cell biology and pathophysiology, being explored as a therapeutic target of clinical interventions against aging and age-related diseases [24]. Studies investigating autophagy in non-model, remarkably long-lived species, like bats, may inform research to modulate autophagy for life extension purposes in humans. In the recent longitudinal study, autophagy was among the GO terms showing positive correlation with age in *M. myotis* blood transcriptomes [9]. Here, we highlight that the strong increase of autophagy-associated GO terms with age in bats, contrasts with decrease observed in human and mouse blood transcriptomes. Many of the identified 26 autophagy genes that positively correlated with age in bats, in contrast to downregulation observed for other mammals, were previously experimentally proven to promote autophagy and directly linked to longevity and age-related diseases by functional studies, e.g. *RB1CC1* [25], *NEDD4* [26], *ULK2* [27], *SIRT1* [28], *DNMIL* [29] and *ATP6V1H* [30].

The age-related increase of autophagy in wild bat blood transcriptomes was mirrored by the increase in marker of autophagy, LC3II/I ratio, observed in skin-derived fibroblasts of *P. kuhlii* and *M. myotis*, which live longer than expected considering their body size. In contrast, skin-derived fibroblasts from the short-lived mouse did not show a similar increase. The data support the hypothesis that bats maintain their ability to ensure cellular homeostasis through efficient removal of cellular damage as they age. It is generally accepted that aging results from the accumulation of cellular damage promoted by chronic stress, and autophagy, being the stress sensor mechanism, attenuates age-associated processes and mediates cytoprotection [31]. In old age, activity of the autophagy machinery is insufficient, either because the autophagic flux is diminished or because there is too much cargo resulting from chronic cellular damage [24]. The increase in expression of autophagy-associated genes and the increased LC3II/I ratio observed in older bats may indicate that their autophagic machinery efficiently removes the higher levels of age-associated cellular damage, which in turn contributes to their longevity. The increased expression of autophagy-associated transcripts with age may be also a compensation response for impaired autophagic degradation (potentially indicated by higher LC3II/I ratio in older animals). However, our experiments with the autophagy inhibitor, bafilomycin A1, do not support this interpretation, at least for *M. myotis*. In contrast to bat species, downregulation of autophagy-associated genes in human and mouse and the lack of increase in LC3II/I ratio in fibroblasts derived from aged mice (22 months, representing approx. 85% of average life span in captivity; [32]) suggests that in mice, the basal autophagic activity is not adjusted to deal with the higher level of age-associated cellular damage. Similarly, the lack of increased autophagy in aged dermal fibroblasts has been recently proposed to contribute to skin aging in humans [33]. Indeed, reduced expression of autophagy-related genes occurs with age and leads to increases in oxidative stress and aberrant protein accumulation in a *Drosophila* model of Alzheimer’s disease [34]. Increased sensitivity of bat autophagy pathways to cellular damage is also plausible in the light of the proposed link between flight and longevity [35, 36]. Flight is associated with a high metabolic rate and an increased risk of oxidative damage, therefore its acquisition may have driven an evolutionary ‘counter-balance’ adaptation of cytoprotective pathways, like autophagy, promoting healthspan extension in bats. Future studies on multiple longer and shorter-lived bat species with varying life history strategies will further explore the role of autophagy in driving bats longevity.

We used a comparative phylogenomics approach to identify the evolutionary adaptations underlying the age-related increase of autophagy activity in bats. Evidence of positive and divergent selection was rare along all tested branches, as expected due to evolutionary constraint on protein-coding genes and in line with the recent large-scale study investigating evolutionary selection in bat lineages that revealed positive selection in <1% of genes investigated [37]. The paucity of positive and divergent selection observed was also expected given conservation of the autophagy pathways across vertebrates [38]. There were, however, several exceptions. Significant amino acid sites showing positive selection were detected along the ancestral bat and ancestral vespertilionid branches for *ATG9B* and *LARP1*, respectively. The ATG9 complex is a multimembrane-spanning autophagy regulator and its expression was also previously reported to reduce the conversion of LC3 [39]. LARP1 stabilizes transcript of *MTOR* [40], which is a potent autophagy inhibitor and a key protein implicated in lifespan regulation [41]. *MTOR* itself, as well as *STOM,* encoding the lysosomal integral membrane protein stomatin [42] and *VPS4A,* involved in lysosomal/endosomal membrane trafficking and autophagosome completion [43] were found to be under divergent selection along the ancestral vespertilionid branch. Intriguingly, *VPS4A* has recently shown a significant correlation with the longevity in the comparative transcriptome study across 16 mammals [44]. *MFN2*, that modulates the ER/mitochondria connections regulating mitochondrial supply of membranes for autophagosome biogenesis [45], showed evidence of sequence divergence in bats relative to other mammals. *MFN2* levels decrease with age and *MFN2* ablation in mice generates a gene signature linked to aging by reducing autophagy [46]. We found evidence of positive selection in *VMP1*, required for autophagosome function [47] and *ZDHHC8*, involved in metabolism pathways [48] in *P. kuhlii*. No evidence of positive selection was found for these genes in the mouse lineage, suggesting a bat-specific pattern of sequence evolution in autophagy-related genes, which may underlie their extended longevity.

Molecular adaptations of the genes identified by our phylogenomic selection pressure tests in the ancestral bat and vespertilionid lineages may drive the age-related increase of autophagic activity in *M. myotis* and *P. kuhlii*. In this study, the *LARP1* and *STOM* are for the first time implicated in an aging context. Our STRING-Protein association network analyses predicted direct interactions between a number of gene products highlighted by our phylogenomic, transcriptomic and cell culture analyses. This suggests functional divergence of the proteins under selection in the bat lineages, implying that they may drive the age-related increase in autophagy signalling observed at the transcriptome and protein level. Further studies are required for functional validation of these findings. For example, knock-ins of bat *ATG9B* or *MFN2* (under selection in ancestral bat lineage) in mouse, or another model species, could be generated to examine the effect of bat-specific adaptations in these genes on the transcriptomic profiles and autophagy activity associated with conditions inducing cellular damage or indeed senescence.

In conclusion, we present an approach based on non-lethal and minimally invasive sampling, offering an unprecedented opportunity to probe the age-dependent autophagy markers in wild transponded bat populations. By integrating comparative cell biology, transcriptomics and genomics, we demonstrated that autophagic activity is enhanced with age in *M. myotis* and *P. kuhlii* bats and uncovered genes under selective pressure that may be responsible for this upregulation.

## MATERIALS AND METHODS

### Comparative transcriptomic analyses of autophagy-associated genes between bat, human and mouse

Previous work from our group [9] suggested that autophagy-related pathways exhibit positive correlation with age in *M. myotis* bats, after correction for sampling site, year of recapture, sequencing bias and individual variation. Seventy genes, which were enriched in the parental GO term ‘autophagy’, were in the module that was positively correlated with age in *M. myotis* bats (FDR < 0.05). To ascertain whether an increase in autophagy-related pathways over age was uniquely seen in bats, here we compared the age-related expression of these 70 autophagy-associated genes across bats, humans and mice using the Huang et al. data sets. The Spearman’s rank correlation coefficients between gene expression and age across taxa were collated (Supplementary Table 6). To investigate the expression pattern at the pathway level, we employed the median of Spearman’s rank correlation coefficients of all 70 genes enriched for the five autophagy related GO terms under the parental term ‘autophagy’ to ascertain their overall pathway expression pattern with age.

### Animals and sampling

All sampling was carried out in accordance with the ethical guidelines in each country (see Supplementary Table 7). The captured individuals were healthy, not showing visible signs of sickness or infection. Wing biopsies were taken from *M. myotis* and *P. kuhlii* wild individuals using a 3 mm biopsy punch (2 per individual). Small clippings (approx. 3 mm wide) were taken from mice ears (C57BL/6J strain) (Supplementary Table 7). All the materials were stored at 4° C in cell culture growth medium (Dulbecco’s MEM high glucose with stabilized glutamine, Biochrom/Merck, 20% FBS, Gibco) supplemented with 1% antibiotics mix (Penicillin-Streptomycin-Fungizone, Lonza BioWhittaker™), and delivered to the lab within 4 days. For the species included in the study, the maximum recorded life span (AnAge), source and age range are detailed in Supplementary Table 7. For each species, the longevity quotient (LQ, observed longevity / expected longevity [49]) was calculated, where expected longevity was obtained using a linear regression fitted to logged values of maximum longevity and mass for all non-flying eutherian species (slope = 0.186, intercept = 0.546) described by Foley et al. [5]. Age described as *n*+ indicates individuals first fitted with transponders as adults *n* years before the subsequent recapture, therefore the true age is unknown. The *n*+ individuals were only included in the oldest age groups (5+ for *M. myotis*, 5+ *for P. kuhlii*) and their age used as *n*+1 for the analyses. Additionally, NHEJ mice flank-skin derived primary fibroblasts were included ([19], Supplementary Table 7).

### Establishment of primary fibroblasts

Primary skin fibroblasts were grown as previously described with some modifications [5]. Wing membrane (bats) and ear (mouse) skin samples were rinsed in fresh growth medium and minced into ~0.5 mm fragments using sterile blade. Skin fragments were resuspended in 3 cm cell culture treated Petri dishes in growth medium supplemented with 0.1 % collagenase type II. After overnight incubation at 37° C, 5% CO_2_, the collagenase was replaced with fresh growth medium. Cells were then fed every 2 – 3 days with growth medium of reduced antibiotic concentration (0.2%). First fibroblast growth was observed after 3 days, and large fibroblast growth halos around fragments of tissue starting to approach one another were obtained after approximately 9-10 days. Typically, cells were passaged using trypsin-EDTA (0.025%) after 10 days of growth and seeded at 100k cells/well in 24-well plates. 24 hrs later, when 80-90% confluent, they were used for experiments.

### Autophagy inducing treatments

Autophagy was induced by 5 hrs of serum deprivation. Where indicated, starvation treatment was performed in the presence of 100 nM bafilomycin A1 (Sigma-Aldrich) to block the autophagosome degradation. Alternatively, autophagy was induced by 5 hrs incubation with 5 μM rapamycin (Cayman Chemical). Following the treatments, cells were washed twice with ice cold PBS and lysed directly in the culture dishes with 55 μl of ice-cold RIPA buffer (20 mM Tris-HCl, pH 7.5; 150 mM NaCl; 1 mM Na2EDTA; 1 mM EGTA; 1% NP-40). After 5 min incubation on ice, the cells were stored at -80° C until western blot analysis.

### Western blot

Total cell lysates were thawed at room temperature for 5 min, transferred to 1.5 ml tubes and mixed with cOmplete™, Mini, EDTA-free protease inhibitor cocktail (Roche) prior to centrifugation for 5 min, 13,000g. The supernatants were mixed 5x Laemmli buffer and a denaturing agent (Fermentas) and run on pre-cast 8-16% polyacrylamide gels (Biorad). Samples were transferred onto PVDF membrane (Biorad), blocked for 1 hr using 2 % non-fat milk in Tris-glycine buffer with 0.1% Tween (TGT), and then incubated overnight with LC3B primary antibody (Cell Signaling #2775) at a 1:500 dilution. The membranes were washed in TGT 3 times and then incubated with goat anti–rabbit secondary antibody (Invitrogen, #65-6120) for 1 hour at a 1:2000 dilution. Following 3 more washes, chemiluminescent detection was carried out using the Western Lightning Plus ECL substrate (PerkinElmer) and images were acquired with the LAS-4000 Image Analyzer (Fujifilm). The membrane was then washed in TGT buffer and then incubated for 1 hour with a GAPDH primary antibody (Cambridge Bioscience #3777R-100). Densitometric analyses of LC3II/I and LC3/GAPDH signal were performed using ImageJ software [50], typically using images taken after 120 s exposure for LC3 staining and 30 s for GAPDH staining. Background subtraction option was used to process images prior to analysis, with options of sliding paraboloid and disabled smoothing. Statistical analyses of the results were performed using SPSS software.

### *P. kuhlii* transcriptome assembly and annotation

Due to lack of genetic resources available for *P. kuhlii* (no genome available at time of analyses and experimentation), we used a transcriptome-based approach to obtain the coding sequences for *P. kuhlii*, required for further phylogenomic analyses. For fibroblast RNA-Seq library preparation, 200k cells/well were seeded in 12-well plates. 24 hrs later, when 80-90% confluent, serum deprivation treatment was performed for 12 hrs. High quality total RNA (RIN scores 9.3-10, 28S/18S 1.9-2.6) was extracted using RNeasy Mini Kit (Qiagen), and DNAse treated with TURBO DNA-free kit (Ambion) according to manufacturers’ instructions. Oligo(dT)s were used to isolate mRNA as part of RNA-Seq library preparation. Paired-end sequencing was performed using the Illumina HiSeq2500 platform (Fasteris) resulting in on average 49.5 million paired-end reads (125 bp) per sample. Raw reads were scanned for adaptor sequences using ‘fastq-mcf’ from the ea-utils package [51]. A minimum base quality score of 25 and a length of 50bp were applied as filtering thresholds across each read. *De novo* assembly methodologies were used to generate the *P. kuhlii* transcriptome assembly [52]. *De novo* assemblies for each sample (3 control and 3 serum starved samples), in addition to a ‘pooled’ super assembly, were generated using Trinity (v2.3.2, [53]). All Trinity outputs were assessed for completeness using Benchmarking Universal Single-Copy Orthologs (BUSCO, [54]) and mammalian orthologs identified in OrthoDB (v9.1; 50 taxa, 4104 BUSCOs, [55]). Coding sequence regions in each assembled transcriptome were identified using FrameDP [56], with predicted peptide sequences less than 20 amino acids removed. Redundant transcripts (100% identical) were identified and removed using cd-hit-est [57], and the transcriptome completeness was further assessed using BUSCO. Blastx [58] was used to map all assembled transcripts to both the TrEMBL and UniProt [59], using an E-value of 1e^-10^, a sequence identity of 80% and a sequence coverage of 70% as thresholds.

### Phylogenomic analyses

### *Gene sequence data*

Genes enriched in the parental GO term ‘autophagy’ were retrieved from *M. myotis* blood transcriptomes (Supplementary Table 6) and autophagy-associated genes were retrieved through a search of gene products associated with the input term “autophagy”, filtered for mammalian genes-only, on the Gene Ontology Consortium open database AmiGO [22] (Supplementary Table 8). This yielded a total of 558 genes after removing redundant names representing the same gene and genes of unknown function. These were used to mine the RefSeq genome annotations [60] of 62 eutherian mammals, representing basal eutherian divergences, including 10 bat species (Supplementary Table 9), using the gene IDs and the CDS mining methodology described in Hughes and Teeling [61]. Genes that were annotated in only 50% or less taxa were excluded from downstream analyses. This operation reduced the number of candidate genes to 406. Assembled RNA transcripts of these target genes were identified in *P. kuhlii* using tblastx [58], with 95% amino acid identity and query genes from *Myotis lucifugus*, *Myotis* *brandtii* and *Myotis davidii* (all *Myotis* species genomes available in Genbank). Removing genes that were not detected in the *P. kuhlii* assembled transcriptome further reduced the final gene number to a total of 274 genes (Supplementary Table 10). Additional taxa whose genomes are highly fragmented and yet to be annotated were mined for target genes using the Hughes and Teeling [61] annotation workflow. This workflow utilizes MAKER [62] with a number of additional pre- and post- annotation steps to recover a gene’s CDS in highly fragmented genome assemblies. As these data were expected to be of lower quality given that they were from low coverage, fragmented genomes, two separate datasets were created: RefSeq only and RefSeq + MAKER output. The RefSeq + MAKER set contained additional sequence data from the all available additional bat genomes (n=5) at the time of analyses (*Rhinolophus ferrumequinum,* *Eidolon helvum, Megaderma* *lyra*, *Pteronotus parnellii*, and a proprietary *Myotis myotis* genome), and three non-chiropteran taxa *Choloepus hoffmanni*, *Procavia capensis* and *Manis pentadactyla* (Supplementary Table 11).

### *Sequence alignment*

All gene files were translated from nucleotide to amino acid sequences and aligned using the phylogeny-aware aligner PRANK [63] with 5 iterations. An in-house Perl script was used to remove poorly aligned regions via Gblocks [64], using the minimum number of sequences allowed for conserved and flank positions, with the output modified alignments subsequently converted into codon alignments. This script is made available on GitHub at https://github.com/batlabucd/GenomeMining. All alignments were converted to PHYLIP format for analyses with PAML [65].

### *Selection tests*

To investigate if selection could be detected in a number of different genes, both the branch-site (positive selection; Model A vs Model A1) and Clade Model C (divergent selection; Clade Model vs M2a_Rel) tests in the PAML package ‘CodeML’ were applied to all alignments for a variety of different ancestral and species-specific lineages. The ancestral bat, ancestral vespertilionid bat, and ancestral *Myotis* bat were designated as foreground branches for calculating omega (ω) (Supplementary Figure 5). We also investigated the selection pressure acting on species-specific lineages: *P. kuhlii* given the quality and availability of transcriptome data and *M. musculus* to compare selection pressures across our experimental taxa. The species tree used in these analyses was created using Meredith et al. [66] for interordinal mammalian relationships, with Foley et al. [5] and Teeling et al. [67] used for bat phylogenies (Supplementary Figure 5). All selection tests were implemented using the Optimised High-throughput Snakemake Automisation of PAML (OHSNAP) pipeline [5]. OHSNAP allows the fully automated execution of a number of CodeML runs in parallel, using multiple models and foreground branches, and was used to run more than 9600 CodeML instances. Likelihood ratio tests (LRTs) were used to compare the fit of the likelihood values from the null and alternative models, with one degree of freedom and *p*-values calculated using a chi-squared distribution. False discovery rate (FDR) correction was applied to *p*-values for each foreground branch and underlying model of selection, with resulting *p*-values above a significance level of 0.05 considered. Sites containing a Bayes Empirical Bayes (BEB) score probability of more than 95% in alignments showing significant differences between null and alternative models were considered to be under selection. These alignments were subsequently visualized to confirm data quality to avoid poorly aligned regions being considered as evidence for selection. Only the genes that had significant signals of selection in both datasets (RefSeq and RefSeq+MAKER) were reported. All steps involved in the phylogenomic analyses are summarized in Supplementary Figure 4. Instances of positive selection in the *P. kuhlii* lineage were further validated against recently published chromosome level genome assemblies made available through the Bat1K project [37].

### Protein-protein interaction network analysis

The interactions between gene products highlighted by cell culture, transcriptomic, phylogenomic analyses in this study were evaluated with the Search Tool for the Retrieval of Interacting Genes/Proteins database, STRING, v.10.5 [23].

### Data availability statement

The *P. kuhlii* fibroblasts transcriptomes generated as part of this analysis will be openly available through the National Center for Biotechnology Information Sequence Reads Archive under accession numbers SRR10129696 - SRR10129701 (BioProject ID: PRJNA565655). All generated nucleotide, protein and codon alignments for autophagy-associated genes under investigation are openly available from: https://figshare.com/s/96e220aba1b424671f5a.

## Supplementary Material

Supplementary Materials

Supplementary Figures

Supplementary Table 1A and 1B

Supplementary Table 2

Supplementary Table 3

Supplementary Table 4

Supplementary Tables 5, 6, and 7

Supplementary Table 8

Supplementary Table 9

Supplementary Table 10

Supplementary Table 11
