# Supplementary material for: Evolution of mammalian longevity: age-related increase in autophagy in bats compared to other mammals
Source: Aging (Albany NY). 2021 Mar 21;13(6):7998–8025. doi: 10.18632/aging.202852 (PMC8034928; doi:10.18632/aging.202852)
Supplement: Supplementary Materials [file aging-13-202852-s001.pdf]

SUPPLEMENTARY MATERIALS

Analysis of starvation-induced differential gene expression of autophagy-associated genes

The *de novo* assembly generated from the pooled transcriptome (3 control and 3 serum starved samples, n=6) was used as a common reference for transcript quantification. For each sample, transcripts were quantified using Salmon (v0.9) [1] with the parameters -*IFS*. Due to higher sensitivity and accuracy, only the protein-coding genes with known functions in the gene-level analysis were investigated. To achieve this, transcripts with the same gene annotation were regarded as different transcripts of the same gene, and their abundance estimates were, accordingly, aggregated to the gene-level using the Bioconductor R package *tximport* [2]. This formed a gene expression matrix, with the horizontal axis representing all 6 samples and the vertical axis representing protein-coding genes. Autophagy-associated genes were identified through a search of gene products associated with the input term "autophagy" on the Gene Ontology Consortium open database AmiGO [3] and by removing redundant names representing the same gene and genes of unknown function. The differential expression analysis of these genes induced by serum starvation was performed using *DESeq2* [4]. The genes with a False Discovery Rate (FDR) < 0.05 were considered significantly differentially expressed (Supplementary Table 2).

REFERENCES

1. Patro R, Duggal G, Love MI, Irizarry RA, Kingsford C. Salmon provides fast and bias-aware quantification of transcript expression. Nat Methods. 2017; 14:417–19. https://doi.org/10.1038/nmeth.4197 PMID:28263959

2. Soneson C, Love MI, Robinson MD. Differential analyses for RNA-seq: transcript-level estimates improve gene-level inferences. F1000Res. 2015; 4:1521. https://doi.org/10.12688/f1000research.7563.2 PMID:26925227

3. Carbon S, Ireland A, Mungall CJ, Shu S, Marshall B, Lewis S; AmiGO Hub; Web Presence Working Group. AmiGO: online access to ontology and annotation data. Bioinformatics. 2009 Jan 15; 25:288–9. https://doi.org/10.1093/bioinformatics/btn615 PMID:19033274

4. Love MI, Huber W, Anders S. Moderated estimation of fold change and dispersion for RNA-seq data with DESeq2. Genome Biol. 2014; 15:550. https://doi.org/10.1186/s13059-014-0550-8 PMID:25516281
