# Supplementary material for: Evolution of mammalian longevity: age-related increase in autophagy in bats compared to other mammals
Source: Aging (Albany NY). 2021 Mar 21;13(6):7998–8025. doi: 10.18632/aging.202852 (PMC8034928; doi:10.18632/aging.202852)
Supplement: Supplementary Figures [file aging-13-202852-s002.pdf]

SUPPLEMENTARY FIGURES

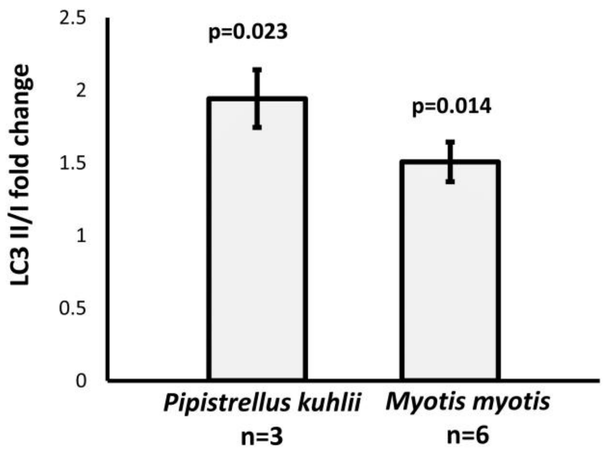

Supplementary Figure 1. Rapamycin-induced change in LC3 II/I ratio in skin-derived fibroblasts from *P. kuhlii* and M. myotis. Data represent mean fold change of LC3 II/I ratio induced by 5μM rapamycin treatment ±SEM (p-values: two-tailed t-test). Individuals tested were 0-3 years old (*P. kuhlii*) and 0-2 years old (*M. myotis*).

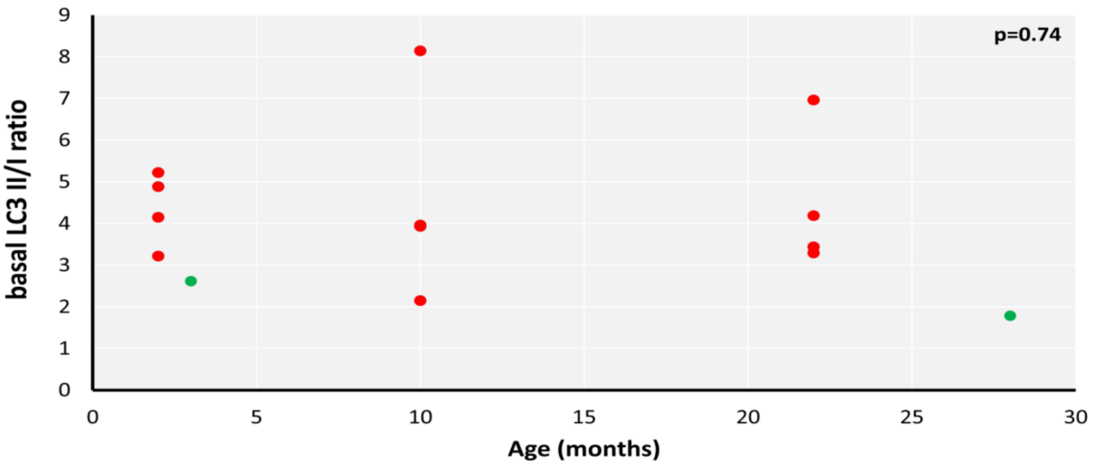

Supplementary Figure 2. Basal LC3 II/I ratio does not increase with age in mouse. In addition to mouse fibroblasts isolated from mouse ear (n=14, red dots), we included the flank skin derived fibroblasts from homozygous NHEJ mouse generated by Vaidya et al., (2014) (n=2, green dots). The LC3II/I ratio was determined as described in experimental procedures and p-value in the top right-hand corner of the plot indicate the significance of linear model.

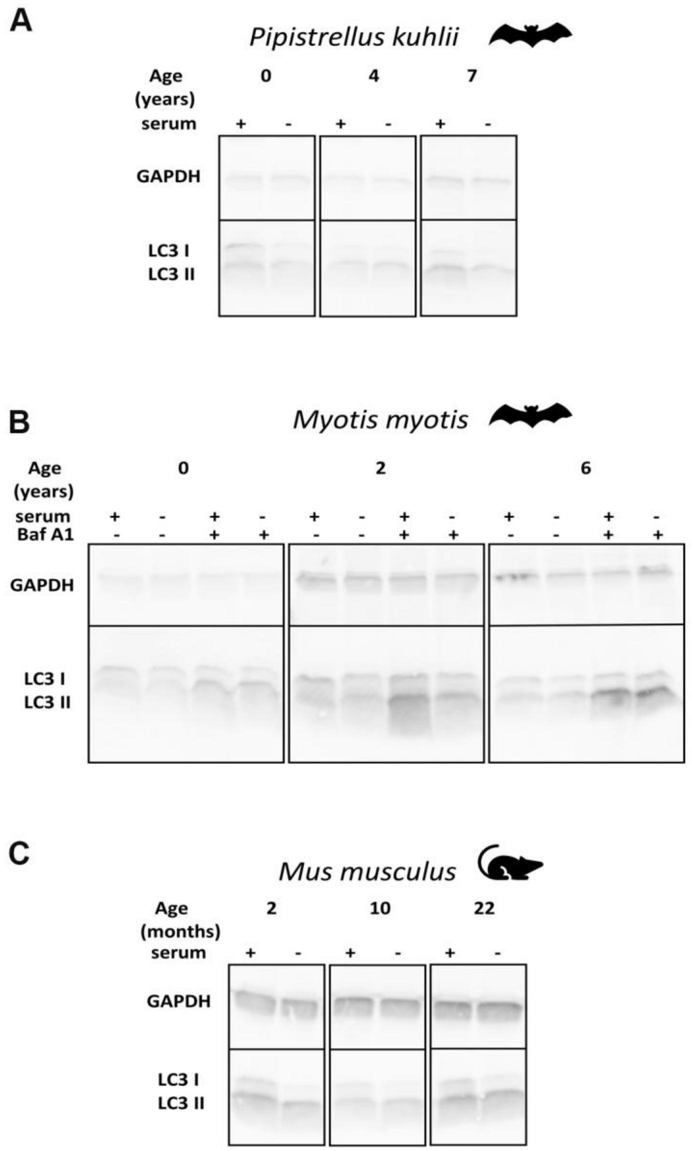

Supplementary Figure 3. Representative Western blot gels for GAPDH and LC3II/LC3I per each species. (A) P. kuhlii, (B) M. myotis and (C) M. musculus.

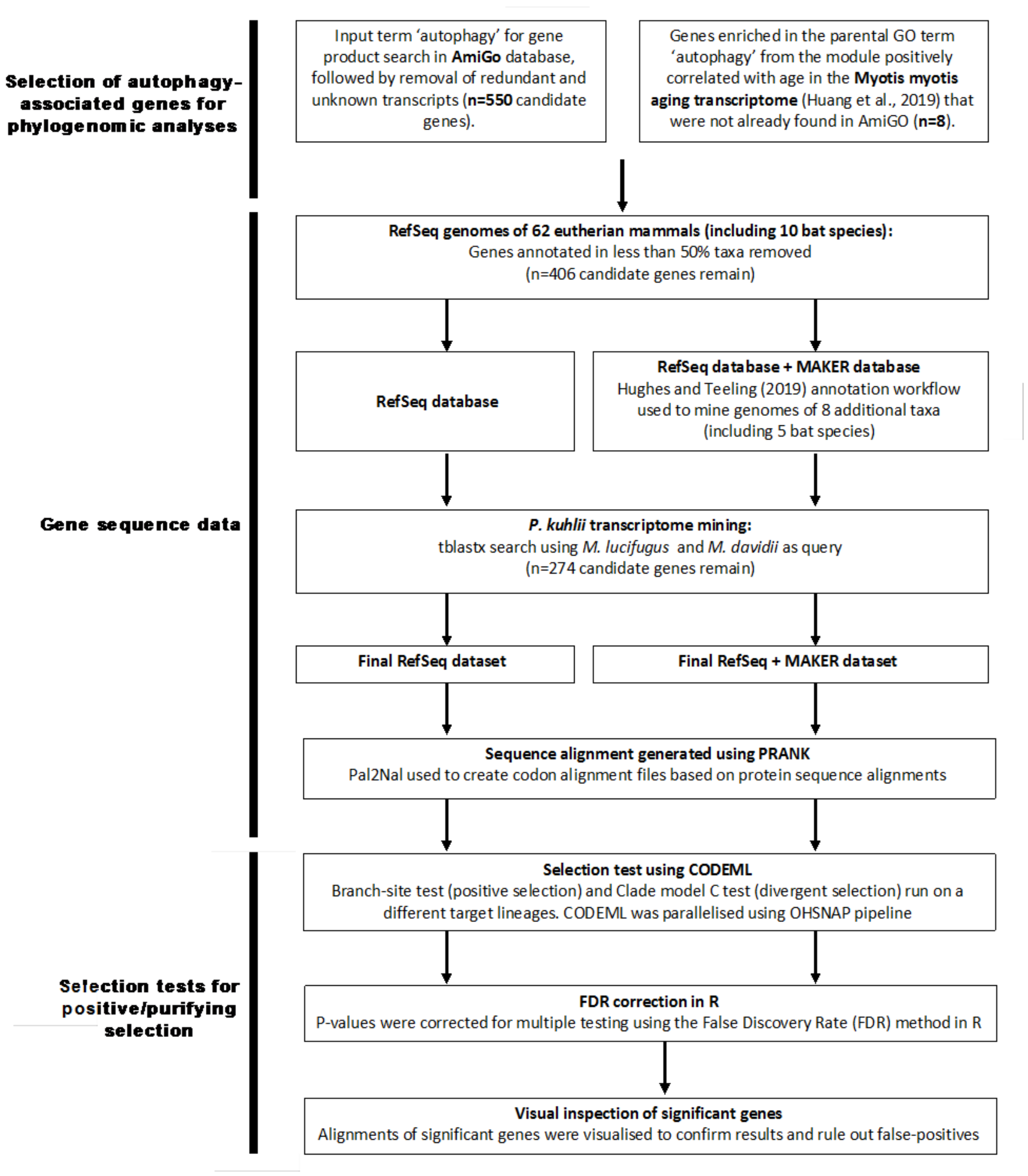

Supplementary Figure 4. Roadmap for phylogenomic analyses performed on autophagy-associated genes across eutherian mammals.

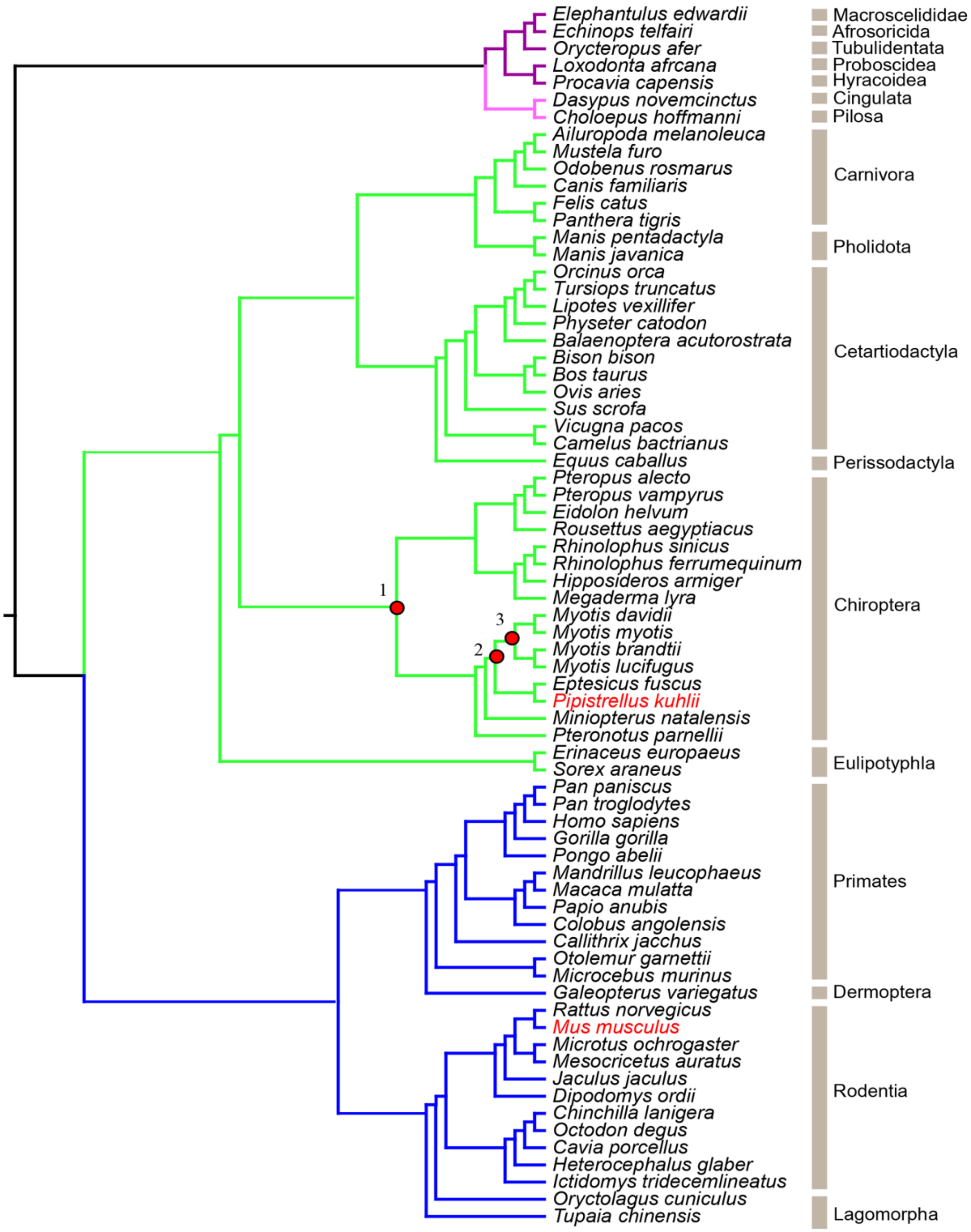

Supplementary Figure 5. Mammalian species tree. Patterns of selection were investigated for in a number of different ancestral bats branches (ancestral bat, ancestral Vespertilionidae and ancestral Myotis branches, labelled node 1, 2 and 3, respectively) and individual lineages (P. kuhlii, M. musculus and M. myotis, highlighted in red). This was done using autophagy-related gene sequences mined from mammalian genomes across all eutherian orders.
