# Supplementary material for: Evolution of mammalian longevity: age-related increase in autophagy in bats compared to other mammals
Source: Aging (Albany NY). 2021 Mar 21;13(6):7998–8025. doi: 10.18632/aging.202852 (PMC8034928; doi:10.18632/aging.202852)
Supplement: Supplementary Table 1A and 1B [file aging-13-202852-s003.pdf]

## SUPPLEMENTARY TABLES

**Supplementary Table 1A. Transcriptome read data.**

| Sample | Status | Number of reads | Number of reads trimmed |
|---|---|---|---|
| Individual 1 | Control | 49,387,054 | 12,904,046 |
| Individual 2 | Control | 52,629,254 | 15,320,180 |
| Individual 3 | Control | 48,344,678 | 13,888,881 |
| **Total** | **Control** | **150,360,986** | **42,113,107** |
| Individual 1 | Starved | 57,071,722 | 12,688,612 |
| Individual 2 | Starved | 47,388,124 | 13,039,158 |
| Individual 3 | Starved | 42,244,472 | 11,607,783 |
| **Total** | **Starved** | **146,704,318** | **37,335,553** |
| Abs. Total | - | 297,065,304 | 79,448,660 |

**Supplementary Table 1B. Transcriptome analyses.**

| Sample | Status | Trinity transcripts | BUSCO % | Complete BUSCOS |
|---|---|---|---|---|
| C13 | Control | 314,290 | 79.9 | 3276 |
| C14 | Control | 294,959 | 79.4 | 3257 |
| C16 | Control | 282,973 | 78.7 | 3232 |
| **Total** | | **892,222** | - | - |
| S13 | Starved | 312,523 | 78.8 | 3237 |
| S14 | Starved | 315,358 | 78 | 3199 |
| S16 | Starved | 269,709 | 77.9 | 3197 |
| **Total** | | **897,590** | - | - |
| Abs. Total | - | 1,789,812 | - | - |
| Pooled | - | 681,843 | 84.2 | 3458 |
| Final | - | 271,767 | 83.6 | 3432 |
