# Supplementary material for: Evolution of mammalian longevity: age-related increase in autophagy in bats compared to other mammals
Source: Aging (Albany NY). 2021 Mar 21;13(6):7998–8025. doi: 10.18632/aging.202852 (PMC8034928; doi:10.18632/aging.202852)
Supplement: Supplementary Table 2 [file aging-13-202852-s004.docx]

| Supplementary Table 2. Differentially expressed (FDR<0.05) autophagy-related genes. | | | |
| --- | --- | --- | --- |
| **Gene** | **log2 (fold change)** | **p-value** | **FDR** |
| *CAPN1* | 0.69944 | 1.16E-23 | 6.24E-21 |
| *Vmp1* | -0.8926 | 2.36E-23 | 6.37E-21 |
| *Ubqln2* | 0.98552 | 5.66E-17 | 1.02E-14 |
| *Pacs2* | 0.61395 | 6.09E-14 | 8.20E-12 |
| *Snrpf* | -1.0383 | 2.78E-12 | 3.00E-10 |
| *CASP3* | -0.6402 | 6.59E-12 | 5.92E-10 |
| *Zdhhc8* | 0.94194 | 7.83E-12 | 6.03E-10 |
| *Srebf1* | 1.40245 | 2.95E-11 | 2.00E-09 |
| *Myom1* | -3.061 | 1.53E-10 | 9.16E-09 |
| *Hsp90aa1* | -0.7178 | 7.67E-10 | 4.14E-08 |
| *Nup93* | -0.6697 | 8.63E-10 | 4.23E-08 |
| *Nrbp2* | 1.06396 | 1.80E-09 | 7.84E-08 |
| *Hmgb1* | -0.665 | 1.89E-09 | 7.84E-08 |
| *Kdr* | 2.98788 | 4.00E-09 | 1.54E-07 |
| *HGF* | -1.8442 | 5.00E-09 | 1.79E-07 |
| *Actl6a* | -0.8306 | 1.51E-08 | 5.10E-07 |
| *Arsa* | 0.62818 | 1.67E-08 | 5.29E-07 |
| *ATP6V1E1* | -0.5314 | 3.57E-08 | 1.07E-06 |
| *Mdh1* | -0.6476 | 4.80E-08 | 1.36E-06 |
| *Rfwd3* | -0.6323 | 7.21E-08 | 1.90E-06 |
| *Eva1b* | 1.03817 | 8.04E-08 | 2.06E-06 |
| *Rnf41* | 0.40983 | 9.96E-08 | 2.44E-06 |
| *Hk2* | -1.1223 | 2.40E-07 | 5.41E-06 |
| *DYNLL1* | -0.6202 | 2.41E-07 | 5.41E-06 |
| *Wbp11* | -0.5058 | 3.11E-07 | 6.70E-06 |
| *Ulk2* | 0.8506 | 3.32E-07 | 6.87E-06 |
| *C9orf72* | -0.7112 | 3.69E-07 | 7.36E-06 |
| *Tfeb* | 0.78518 | 6.45E-07 | 1.24E-05 |
| *MT-III* | -0.6287 | 6.82E-07 | 1.27E-05 |
| *Rnf185* | 0.54448 | 7.38E-07 | 1.33E-05 |
| *Anxa7* | 0.79869 | 1.46E-06 | 2.54E-05 |
| *Anxa5* | 0.29805 | 2.71E-06 | 4.57E-05 |
| *Atg2a* | 0.59084 | 4.81E-06 | 7.85E-05 |
| *Mrps2* | 0.69439 | 6.85E-06 | 0.00011 |
| *Vcp* | -0.5353 | 1.08E-05 | 0.00017 |
| *Atm* | -0.5527 | 1.10E-05 | 0.00017 |
| *Phyhip* | 1.784 | 1.22E-05 | 0.00018 |
| *VPS37C* | 0.55702 | 1.31E-05 | 0.00019 |
| *PSAP* | 0.54436 | 1.56E-05 | 0.00022 |
| *GAPDH* | -0.7456 | 1.76E-05 | 0.00024 |
| *Snrpb2* | -0.653 | 2.10E-05 | 0.00028 |
| *Fancc* | -0.9352 | 2.30E-05 | 0.0003 |
| *Snrpb* | -0.4818 | 3.31E-05 | 0.00041 |
| *Atg4a* | -0.4445 | 3.72E-05 | 0.00046 |
| *ATP6V1D* | -0.5587 | 4.82E-05 | 0.00058 |
| *Bok* | 1.42635 | 5.29E-05 | 0.00062 |
| *Chaf1b* | -0.8215 | 7.36E-05 | 0.00084 |
| *Pik3r2* | 0.45427 | 7.73E-05 | 0.00087 |
| *Bloc1s1* | 0.51286 | 8.02E-05 | 0.00088 |
| *MVB12A* | 0.56489 | 8.87E-05 | 0.00095 |
| *Mtdh* | -0.3593 | 9.01E-05 | 0.00095 |
| *Slc17a9* | 0.80798 | 9.50E-05 | 0.00098 |
| *Wipi2* | 0.32483 | 0.00012 | 0.00123 |
| *Ctsa* | 0.51709 | 0.00013 | 0.00134 |
| *Rab3gap2* | 0.45139 | 0.00017 | 0.00166 |
| *Tigar* | -0.7358 | 0.0002 | 0.00191 |
| *Usp13* | -0.6239 | 0.0002 | 0.00193 |
| *MAP1S* | 0.53331 | 0.00021 | 0.00194 |
| *Bcl2* | 1.52436 | 0.00024 | 0.0022 |
| *PRKAG3* | 4.60023 | 0.00024 | 0.0022 |
| *Tecpr1* | 0.48 | 0.00026 | 0.0023 |
| *Cspg5* | 1.30087 | 0.0003 | 0.0026 |
| *Htt* | 0.4166 | 0.00031 | 0.00268 |
| *Tcirg1* | 0.41309 | 0.00035 | 0.00297 |
| *MFN1* | -0.4248 | 0.00036 | 0.00297 |
| *Rab33b* | -0.4179 | 0.00046 | 0.00374 |
| *Atg13* | 0.42668 | 0.00049 | 0.00396 |
| *ATP6V1G1* | -0.2567 | 0.0005 | 0.00396 |
| *Map2k1* | -0.7412 | 0.00056 | 0.00436 |
| *Zcchc17* | -0.5911 | 0.0006 | 0.0046 |
| *Dap* | 0.20966 | 0.00069 | 0.00522 |
| *Mtmr3* | 0.3076 | 0.00073 | 0.00546 |
| *CHMP2B* | -0.46 | 0.00095 | 0.00696 |
| *Hif1a* | -0.6319 | 0.00096 | 0.00696 |
| *SNX5* | -0.3476 | 0.00097 | 0.00698 |
| *Cisd2* | -0.3792 | 0.00099 | 0.00702 |
| *Atg16l1* | -0.2809 | 0.0012 | 0.00828 |
| *Prkd2* | 0.50303 | 0.00121 | 0.00828 |
| *Wipi1* | 0.79189 | 0.00121 | 0.00828 |
| *Vipas39* | -0.4711 | 0.00126 | 0.00851 |
| *VTA1* | -0.505 | 0.00131 | 0.00867 |
| *Atg5* | -0.3765 | 0.00132 | 0.00867 |
| *Cln3* | 0.45382 | 0.00147 | 0.00958 |
| *ATP6V1C1* | -0.3452 | 0.00151 | 0.00969 |
| *Ist1* | -0.3232 | 0.00154 | 0.00975 |
| *Pex3* | -0.4387 | 0.00162 | 0.01016 |
| *Lsm4* | -0.3826 | 0.00182 | 0.0113 |
| *Nod1* | 0.72473 | 0.00188 | 0.01149 |
| *Sqstm1* | 0.46392 | 0.00196 | 0.01188 |
| *Ehmt2* | 0.27706 | 0.0022 | 0.01319 |
| *Atp13a2* | 0.4485 | 0.00226 | 0.0134 |
| *Tp53* | -0.603 | 0.00234 | 0.01368 |
| *STAM2* | 0.53481 | 0.00238 | 0.01368 |
| *Hspa8* | -0.312 | 0.00239 | 0.01368 |
| *TSG101* | -0.3216 | 0.00244 | 0.01387 |
| *TBC1D12* | 0.52341 | 0.0025 | 0.01398 |
| *Obscn* | 3.28386 | 0.00252 | 0.01398 |
| *TRIM22* | 0.92837 | 0.0031 | 0.01703 |
| *Trim65* | 0.73605 | 0.00317 | 0.01728 |
| *Rpl28* | 0.52742 | 0.00321 | 0.0173 |
| *Krt15* | 3.35447 | 0.00337 | 0.01798 |
| *Zc3h12a* | 0.53068 | 0.00354 | 0.01858 |
| *Sirt1* | -0.3969 | 0.00355 | 0.01858 |
| *DAPK1* | 0.90465 | 0.00362 | 0.01875 |
| *Itpkc* | 0.6073 | 0.00367 | 0.01885 |
| *Ift20* | -0.4583 | 0.00383 | 0.01949 |
| *Rasip1* | 0.4009 | 0.00433 | 0.02183 |
| *TOMM70* | -0.3704 | 0.00443 | 0.02209 |
| *TAB2* | 0.40585 | 0.00449 | 0.02218 |
| *Scoc* | -0.4682 | 0.00453 | 0.02218 |
| *HGS* | 0.33151 | 0.00457 | 0.02218 |
| *Ruvbl1* | -0.3773 | 0.00464 | 0.02231 |
| *Chmp4b* | -0.1855 | 0.00522 | 0.02492 |
| *Park2* | 0.8393 | 0.00533 | 0.02521 |
| *TOMM20* | -0.3897 | 0.00543 | 0.02545 |
| *Pafah1b2* | -0.3074 | 0.00598 | 0.02778 |
| *TOMM5* | -0.4495 | 0.00627 | 0.02888 |
| *Rbm18* | -0.2979 | 0.00635 | 0.02899 |
| *Trim8* | 0.54653 | 0.00694 | 0.03142 |
| *Mtcl1* | 0.65743 | 0.00734 | 0.03299 |
| *OSBPL7* | 0.33652 | 0.00743 | 0.0331 |
| *Dear1* | 0.39277 | 0.00798 | 0.03525 |
| *VPS25* | -0.3175 | 0.0082 | 0.03568 |
| *Gba* | 0.41369 | 0.00821 | 0.03568 |
| *Usp33* | -0.2893 | 0.00889 | 0.03835 |
| *Vps33b* | 0.2286 | 0.01041 | 0.04453 |
| *TECPR2* | 0.38955 | 0.01078 | 0.04575 |
| *Fnbp1l* | -0.6311 | 0.01086 | 0.04575 |
| *Alkbh5* | 0.35737 | 0.01112 | 0.04645 |
| *DZANK1* | -0.5923 | 0.01127 | 0.04672 |
| *Lamp1* | 0.32951 | 0.01194 | 0.04915 |
