# Supplementary material for: Evolution of mammalian longevity: age-related increase in autophagy in bats compared to other mammals
Source: Aging (Albany NY). 2021 Mar 21;13(6):7998–8025. doi: 10.18632/aging.202852 (PMC8034928; doi:10.18632/aging.202852)
Supplement: Supplementary Table 3 [file aging-13-202852-s005.pdf]

Supplementary Table 3. Codeml results.

Model	Gene	Dataset	Site(s) in alignment	Corresponding sites in amino acid sequence	FDR p-value	ω
			**Ancestral bat lineage**			
Branch-site	*ATG9B*	RefSeq	60	60V (human: 59S)	0.009	Fg 443.74
		RefSeq + MAKER	60		0.004	Fg 434.69
Clade Model C	*MFN2*	RefSeq	-	-	0.002	Fg 3.38<br>Bg 0.19
		RefSeq + MAKER	-	-	0.035	Fg 2.35<br>Bg 0.20
			***Vespertillionidae* ancestral lineage**			
Branch-site	*LARP1*	RefSeq	574	605N (human 684F)	0.008	Fg 58.87
		RefSeq + MAKER	546	605N (human 684F)	0.001	Fg 61.62
Clade Model C	*MTOR*	RefSeq	-	-	0.007	Fg 1.41<br>Bg 0.21
		RefSeq + MAKER	-	-	0.015	Fg 1.39<br>Bg 0.21
	NPC1	RefSeq	-	-	0.004	Fg 0.12<br>Bg 0.24
		RefSeq + MAKER	-	-	0.002	Fg 0.11<br>Bg 0.25
	STOM	RefSeq	-	-	0.0004	Fg 2.53<br>Bg 0.17
		RefSeq + MAKER	-	-	0.0006	Fg 2.41<br>Bg 0.18
	VPS4A	RefSeq	-	-	0.003	Fg 1.4<br>Bg 0.15
		RefSeq + MAKER	-	-	0.002	Fg 1.57<br>Bg 0.14
			Pipistrellus kuhlli			
Branch-site	*VMP1*	RefSeq	No BEB sites	-	0.017	Fg: 999
		RefSeq + MAKER	170	170P (human 170D)	0.025	Fg: 999
	ZDHHC8	RefSeq	588	585A (human 591S)	0.017	Fg: 120.36
		RefSeq + MAKER	588, 754	585A (human 591S), 723R (human 760A)	0.026	Fg: 129.48
Clade Model C	*SFRP4*	RefSeq	-	-	0.003	Fg 1.29<br>Bg 0.21
		RefSeq + MAKER	-	-	0.003	Fg 1.32<br>Bg 0.21
			Mus musculus			
Branch-site	*PSAP*	RefSeq	248, 471	251C (human 252A), 506G (human 476S)	0.001	Fg: 123.42
		RefSeq + MAKER	248, 468	251C (human 252A), 506G (human 476S)	0.006	Fg: 123.34

Clade Model C	*SNX14*	RefSeq	-	-	0.04	Fg 0.00<br>Bg 0.18
		RefSeq + MAKER	-	-	0.013	Fg 0.00<br>Bg 0.19

Fg = Foreground, Bg = Background. Bat lineage-specific sites under positive selection are reported with reference to corresponding amino acid in human CDS. Sites showing significant selection identified in optimized alignments and their corresponding amino acid position are shown.
