# Supplementary material for: Evolution of mammalian longevity: age-related increase in autophagy in bats compared to other mammals
Source: Aging (Albany NY). 2021 Mar 21;13(6):7998–8025. doi: 10.18632/aging.202852 (PMC8034928; doi:10.18632/aging.202852)
Supplement: Supplementary Table 4 [file aging-13-202852-s006.docx]

Supplementary Table 4. Functional enrichment in Biological Process GO terms of the STRING protein-protein interaction network including genes under selective pressure in bat lineages, genes showing bat-specific upregulation with age, and LC3.

| **Pathway ID** | **Pathway Description** | **Observed Gene Count** | **FDR** | **Matching Protein in the Network** |
| --- | --- | --- | --- | --- |
| GO.0010506 | regulation of autophagy | 13 | 1.06E-13 | Dnm1l,Larp1,Mtor,Npc1,Nrbf2,Pafah1b2,Pik3ca,Rb1cc1,Sh3glb1,Sirt1,Stom,Ulk2,Usp33 |
| GO.0031669 | cellular response to nutrient levels | 12 | 1.06E-13 | Atg9b,Eif2ak4,Map1lc3a,Mtor,Pik3c3,Pik3r4,Rb1cc1,Sh3glb1,Sirt1,Ubxn2b,Ulk2,Usp33 |
| GO.0009267 | cellular response to starvation | 11 | 4.28E-13 | Atg9b,Eif2ak4,Map1lc3a,Pik3c3,Pik3r4,Rb1cc1,Sh3glb1,Sirt1,Ubxn2b,Ulk2,Usp33 |
| GO.0006914 | autophagy | 10 | 8.37E-12 | Atg9b,Map1lc3a,Mfn2,Npc1,Nrbf2,Pik3c3,Pik3r4,Rb1cc1,Ubxn2b,Ulk2 |
| GO.0009894 | regulation of catabolic process | 14 | 1.93E-10 | Csnk2a1,Dnm1l,Larp1,Nedd4,Npc1,Nrbf2,Pafah1b2,Pik3ca,Rb1cc1,Sh3glb1,Sirt1,Stom,Ulk2,Usp33 |
| GO.0031329 | regulation of cellular catabolic process | 13 | 4.78E-10 | Csnk2a1,Dnm1l,Larp1,Npc1,Nrbf2,Pafah1b2,Pik3ca,Rb1cc1,Sh3glb1,Sirt1,Stom,Ulk2,Usp33 |
| GO.0016241 | regulation of macroautophagy | 9 | 4.99E-09 | Dnm1l,Larp1,Mtor,Npc1,Pafah1b2,Pik3ca,Sh3glb1,Sirt1,Stom |
| GO.0033554 | cellular response to stress | 16 | 4.99E-09 | Atg9b,Eif2ak4,Map1lc3a,Mtor,Nedd4,Nrbf2,Pik3c3,Pik3ca,Pik3r4,Rb1cc1,Sh3glb1,Sirt1,Stom,Ubxn2b,Ulk2,Usp33 |
| GO.0016236 | macroautophagy | 7 | 7.01E-09 | Atg9b,Map1lc3a,Pik3c3,Pik3r4,Rb1cc1,Ubxn2b,Ulk2 |
| GO.0000045 | autophagosome assembly | 6 | 3.27E-08 | Atg9b,Map1lc3a,Pik3c3,Rb1cc1,Ubxn2b,Ulk2 |
| GO.0009056 | catabolic process | 15 | 5.44E-08 | Atg9b,Map1lc3a,Mfn2,Npc1,Nrbf2,Pafah1b2,Pik3c3,Pik3r4,Rb1cc1,Sh3glb1,Sirt1,Ubxn2b,Ulk2,Usp33, Vps4a |
| GO.0044248 | cellular catabolic process | 14 | 6.54E-08 | Atg9b,Map1lc3a,Mfn2,Npc1,Nrbf2,Pik3c3,Pik3r4,Rb1cc1,Sh3glb1,Sirt1,Ubxn2b,Ulk2,Usp33,Vps4a |
| GO.0007033 | vacuole organization | 7 | 6.72E-08 | Atg9b,Map1lc3a,Pik3c3,Rb1cc1,Ubxn2b,Ulk2,Vps4a |
| GO.0007154 | cell communication | 21 | 0.00000034 | Atg9b,Csnk2a1,Dnm1l,Eif2ak4,Larp1,Map1lc3a,Map3k7,Mtor,Nedd4,Npc1,Pik3c3,Pik3ca,Pik3r4,Rab3gap1,Rb1cc1,Rock1,Sh3glb1,Sirt1,Ubxn2b,Ulk2,Usp33 |
| GO.0032107 | regulation of response to nutrient levels | 8 | 3.61E-07 | Dnm1l,Larp1,Npc1,Pafah1b2,Pik3ca,Sh3glb1,Sirt1,Stom |
| GO.0006950 | response to stress | 18 | 5.84E-07 | Atg9b,Eif2ak4,Map1lc3a,Mfn2,Mtor,Nedd4,Nrbf2,Pik3c3,Pik3ca,Pik3r4,Rb1cc1,Sh3glb1,Sirt1,Stom,Tbk1, Ubxn2b,Ulk2,Usp33 |
| GO.0051716 | cellular response to stimulus | 22 | 0.00000166 | Atg9b,Csnk2a1,Dnm1l,Eif2ak4,Larp1,Map1lc3a,Map3k7,Mtor,Nedd4,Nrbf2,Pik3c3,Pik3ca,Pik3r4,Rab3gap1,Rb1cc1,Rock1,Sh3glb1,Sirt1,Stom,Ubxn2b,Ulk2,Usp33 |
| GO.0061024 | membrane organization | 10 | 0.00000228 | Atg9b,Dnm1l,Map1lc3a,Mfn2,Npc1,Rab3gap1,Sh3glb1,Ubxn2b,Vps4a,Vta1 |
| GO.0006468 | protein phosphorylation | 11 | 0.0000025 | Csnk2a1,Eif2ak4,Map3k7,Mtor,Pik3c3,Pik3ca,Pik3r4,Rb1cc1,Rock1,Tbk1,Ulk2 |
| GO.0009605 | response to external stimulus | 14 | 0.00000251 | Atg9b,Eif2ak4,Map1lc3a,Mtor,Pik3c3,Pik3ca,Pik3r4,Rb1cc1,Sh3glb1,Sirt1,Tbk1,Ubxn2b,Ulk2,Usp33 |
| GO.0044267 | cellular protein metabolic process | 18 | 0.00000251 | Csnk2a1,Eif2ak4,Larp1,Map3k7,Mtor,Npc1,Pik3c3,Pik3ca,Pik3r4,Rb1cc1,Rock1,Sh3glb1,Sirt1,Tbk1,Ubxn2b Ulk2,Usp33,Vps4a |
| GO.0080135 | regulation of cellular response to stress | 10 | 0.00000436 | Dnm1l,Larp1,Map3k7,Mtor,Npc1,Pafah1b2,Pik3ca,Sh3glb1,Sirt1,Stom |
| GO.0010647 | positive regulation of cell communication | 13 | 0.00000977 | Csnk2a1,Dnm1l,Larp1,Map3k7,Mtor,Nedd4,Pafah1b2,Pik3ca,Rab3gap1,Sh3glb1,Sirt1,Stom,Tbk1 |
| GO.0008152 | metabolic process | 27 | 0.0000147 | Atg9b,Atp6v1c1,Atp6v1h,Csnk2a1,Dnm1l,Eif2ak4,Larp1,Map1lc3a,Map3k7,Mfn2,Mtor,Npc1,Nrbf2,Pafah1b2,Pik3ca,Pik3r4,Rb1cc1,Rock1,Sh3glb1,Sirt1,Tbk1,U2af1,Ubxn2b,Ulk2,Usp33,Vps36,Vps4a |
| GO.0009896 | positive regulation of catabolic process | 8 | 0.0000164 | Csnk2a1,Larp1,Nedd4,Pafah1b2,Pik3ca,Sh3glb1,Sirt1,Stom |
| GO.0010646 | regulation of cell communication | 16 | 0.0000174 | Csnk2a1,Dnm1l,Larp1,Map3k7,Mfn2,Npc1,Pafah1b2,Pik3ca,Rab3gap1,Rb1cc1,Sh3glb1,Sirt1,Snx14,Stom, Tbk1,Usp33 |
| GO.0016239 | positive regulation of macroautophagy | 6 | 0.0000185 | Larp1,Pafah1b2,Pik3ca,Sh3glb1,Sirt1,Stom |
| GO.0006464 | cellular protein modification process | 15 | 0.0000381 | Csnk2a1,Eif2ak4,Map3k7,Mtor,Nedd4,Npc1,Pik3c3,Pik3ca,Pik3r4,Rb1cc1,Rock1,Sirt1,Tbk1,Ulk2,Usp33 |
| GO.0080134 | regulation of response to stress | 11 | 0.0000381 | Dnm1l,Larp1,Map3k7,Mtor,Npc1,Pafah1b2,Pik3ca,Sh3glb1,Sirt1,Stom,Tbk1 |
| GO.0050896 | response to stimulus | 22 | 0.0000446 | Atg9b,Csnk2a1,Dnm1l,Eif2ak4,Larp1,Map1lc3a,Map3k7,Mfn2,Mtor,Nrbf2,Pik3ca,Pik3r4,Rab3gap1,Rb1cc1,Rock1,Sh3glb1,Sirt1,Stom,Tbk1,Ubxn2b,Ulk2,Usp33 |
| GO.0044085 | cellular component biogenesis | 13 | 0.0000623 | Atg9b,Dnm1l,Map1lc3a,Pik3c3,Rb1cc1,Rock1,Sh3glb1,Sirt1,Stom,Ubxn2b,Ulk2,Vps4a,Vta1 |
| GO.0044237 | cellular metabolic process | 24 | 0.0000897 | Atg9b,Csnk2a1,Eif2ak4,Larp1,Map1lc3a,Map3k7,Mfn2,Mtor,Npc1,Nrbf2,Pik3c3,Pik3ca,Pik3r4,Rb1cc1,Rock1,Sh3glb1,Sirt1,Tbk1,U2af1,Ubxn2b,Ulk2,Usp33,Vps36,Vps4a |
| GO.0008104 | protein localization | 12 | 0.00009 | Atg9b,Dnm1l,Mfn2,Nedd4,Npc1,Pik3r4,Rab3gap1,Sh3glb1,Snx14,Vps36,Vps4a,Vta1 |
| GO.0006996 | organelle organization | 15 | 0.0000907 | Atg9b,Dnm1l,Map1lc3a,Mfn2,Pik3r4,Rab3gap1,Rb1cc1,Rock1,Sh3glb1,Sirt1,Ubxn2b,Ulk2,Usp33,Vps4a, Vta1 |
| GO.0044802 | single-organism membrane organization | 8 | 0.000109 | Atg9b,Dnm1l,Mfn2,Npc1,Rab3gap1,Sh3glb1,Ubxn2b,Vps4a |
| GO.0019538 | protein metabolic process | 17 | 0.000141 | Csnk2a1,Eif2ak4,Larp1,Map3k7,Mtor,Npc1,Pik3ca,Pik3r4,Rb1cc1,Rock1,Sh3glb1,Sirt1,Tbk1,Ubxn2b,Ulk2,Usp33,Vps4a |
| GO.0022607 | cellular component assembly | 12 | 0.000141 | Atg9b,Dnm1l,Map1lc3a,Pik3c3,Rb1cc1,Rock1,Sh3glb1,Stom,Ubxn2b,Ulk2,Vps4a,Vta1 |
| GO.0061025 | membrane fusion | 5 | 0.000166 | Dnm1l,Map1lc3a,Mfn2,Ubxn2b,Vps4a |
| GO.0006796 | phosphate-containing compound metabolic process | 12 | 0.000211 | Csnk2a1,Eif2ak4,Map3k7,Mtor,Pik3c3,Pik3ca,Pik3r4,Rb1cc1,Rock1,Sh3glb1,Tbk1,Ulk2 |
| GO.0016043 | cellular component organization | 18 | 0.000211 | Atg9b,Dnm1l,Map1lc3a,Mfn2,Mtor,Npc1,Pik3r4,Rab3gap1,Rb1cc1,Rock1,Sh3glb1,Sirt1,Stom,Ubxn2b,Ulk2, Usp33,Vps4a,Vta1 |
| GO.0033043 | regulation of organelle organization | 10 | 0.000211 | Csnk2a1,Dnm1l,Mtor,Pik3ca,Rab3gap1,Rock1,Sh3glb1,Sirt1,Stom,Vps4a |
| GO.0044260 | cellular macromolecule metabolic process | 21 | 0.000211 | Csnk2a1,Eif2ak4,Larp1,Map3k7,Mtor,Npc1,Nrbf2,Pik3c3,Pik3ca,Pik3r4,Rb1cc1,Rock1,Sh3glb1,Sirt1,Tbk1,U2af1,Ubxn2b,Ulk2,Usp33,Vps36,Vps4a |
| GO.0010638 | positive regulation of organelle organization | 8 | 0.000239 | Dnm1l,Mtor,Pik3ca,Rab3gap1,Sh3glb1,Sirt1,Stom,Vps4a |
| GO.0009987 | cellular process | 29 | 0.000269 | Atg9b,Atp6v1h,Csnk2a1,Dnm1l,Eif2ak4,Larp1,Map1lc3a,Map3k7,Mfn2,Mtor,Npc1,Nrbf2,Pafah1b2,Pik3ca,Pik3r4,Rab3gap1,Rb1cc1,Rock1,Sh3glb1,Sirt1,Stom,Tbk1,U2af1,Ubxn2b,Ulk2,Usp33,Vps36,Vps4a,Vta1 |
| GO.0032465 | regulation of cytokinesis | 4 | 0.00032 | Pik3c3,Pik3r4,Sh3glb1,Vps4a |
| GO.0048583 | regulation of response to stimulus | 15 | 0.000342 | Csnk2a1,Dnm1l,Larp1,Map3k7,Mfn2,Npc1,Pafah1b2,Pik3ca,Rab3gap1,Rb1cc1,Sh3glb1,Sirt1,Snx14,Stom,Usp33 |
| GO.0032801 | receptor catabolic process | 3 | 0.000374 | Nedd4,Pik3r4,Sh3glb1 |
| GO.0090148 | membrane fission | 2 | 0.000472 | Dnm1l, Sh3glb1 |
| GO.0044712 | single-organism catabolic process | 8 | 0.00056 | Atg9b,Map1lc3a,Pafah1b2,Pik3c3,Pik3r4,Rb1cc1,Ubxn2b,Ulk2 |
| GO.0044710 | single-organism metabolic process | 16 | 0.00059 | Atg9b,Eif2ak4,Map1lc3a,Map3k7,Mtor,Nedd4,Npc1,Pafah1b2,Pik3c3,Pik3ca,Pik3r4,Rb1cc1,Sh3glb1,Sirt1,Ubxn2b,Ulk2 |
| GO.0016192 | vesicle-mediated transport | 9 | 0.000659 | Atp6v1h,Dnm1l,Nedd4,Npc1,Pik3c3,Rab3gap1,Usp33,Vps4a,Vta1 |
| GO.0044403 | symbiosis, encompassing mutualism through parasitism | 5 | 0.00093 | Nedd4,Npc1,Stom,Vps4a,Vta1 |
| GO.0030242 | pexophagy | 2 | 0.00124 | Pik3c3,Pik3r4 |
| GO.0065009 | regulation of molecular function | 13 | 0.00124 | Csnk2a1,Map3k7,Mtor,Nedd4,Nrbf2,Pik3ca,Pik3r4,Rock1,Sh3glb1,Sirt1,Stom,Ubxn2b,Usp33 |
| GO.0048584 | positive regulation of response to stimulus | 11 | 0.00125 | Csnk2a1,Dnm1l,Larp1,Map3k7,Mtor,Nedd4,Pafah1b2,Pik3ca,Sh3glb1,Sirt1,Stom |
| GO.0072665 | protein localization to vacuole | 3 | 0.00142 | Nedd4,Pik3r4,Sh3glb1 |
| GO.0010648 | negative regulation of cell communication | 9 | 0.00148 | Map3k7,Mfn2,Mtor,Nedd4,Npc1,Rb1cc1,Sh3glb1,Sirt1,Snx14 |
| GO.0042149 | cellular response to glucose starvation | 3 | 0.00172 | Pik3c3,Pik3r4,Sh3glb1 |
| GO.0051128 | regulation of cellular component organization | 12 | 0.00172 | Csnk2a1,Dnm1l,Map3k7,Mtor,Nedd4,Pik3ca,Rab3gap1,Sh3glb1,Sirt1,Stom,Ulk2,Vps4a |
| GO.0009966 | regulation of signal transduction | 12 | 0.00191 | Csnk2a1,Dnm1l,Map3k7,Mfn2,Mtor,Rab3gap1,Rb1cc1,Sh3glb1,Sirt1,Snx14,Tbk1,Usp33 |
| GO.0045184 | establishment of protein localization | 9 | 0.00191 | Mfn2,Nedd4,Npc1,Pik3r4,Rab3gap1,Snx14,Vps36,Vps4a,Vta1 |
| GO.0051641 | cellular localization | 11 | 0.00191 | Atg9b,Dnm1l,Mfn2,Nedd4,Npc1,Pik3c3,Pik3r4,Rab3gap1,Sh3glb1,Vps36,Vps4a |
| GO.0060341 | regulation of cellular localization | 9 | 0.00191 | Dnm1l,Mtor,Nedd4,Pik3c3,Rab3gap1,Sh3glb1,Sirt1,Stom,Vps4a |
| GO.0007031 | peroxisome organization | 3 | 0.00199 | Dnm1l,Pik3c3,Pik3r4 |
| GO.0033365 | protein localization to organelle | 6 | 0.00199 | Dnm1l,Mfn2,Nedd4,Pik3r4,Rab3gap1,Sh3glb1 |
| GO.0051130 | positive regulation of cellular component organization | 9 | 0.00205 | Dnm1l,Mtor,Pik3ca,Rab3gap1,Rock1,Sh3glb1,Sirt1,Stom,Vps4a |
| GO.0060548 | negative regulation of cell death | 8 | 0.00212 | Csnk2a1,Map3k7,Npc1,Pik3ca,Rb1cc1,Rock1,Sh3glb1,Sirt1 |
| GO.0006897 | endocytosis | 6 | 0.00226 | Atp6v1h,Dnm1l,Nedd4,Npc1,Pik3c3,Usp33 |
| GO.0031323 | regulation of cellular metabolic process | 18 | 0.00245 | Csnk2a1,Dnm1l,Eif2ak4,Larp1,Map3k7,Npc1,Nrbf2,Pafah1b2,Pik3ca,Pik3r4,Rb1cc1,Sh3glb1,Sirt1,Stom, Tbk1,Ulk2,Usp33,Vps36 |
| GO.0044699 | single-organism process | 26 | 0.00255 | Atg9b,Atp6v1h,Csnk2a1,Dnm1l,Eif2ak4,Larp1,Map1lc3a,Map3k7,Mfn2,Mtor,Pafah1b2,Pik3c3,Pik3ca,Pik3r4,Rab3gap1,Rb1cc1,Rock1,Sh3glb1,Sirt1,Tbk1,Ubxn2b,Ulk2,Usp33,Vps36,Vps4a,Vta1 |
| GO.0043170 | macromolecule metabolic process | 20 | 0.00264 | Csnk2a1,Eif2ak4,Larp1,Map3k7,Mtor,Npc1,Nrbf2,Pik3ca,Pik3r4,Rb1cc1,Rock1,Sh3glb1,Sirt1,Tbk1,U2af1,Ubxn2b,Ulk2,Usp33,Vps36,Vps4a |
| GO.0051179 | localization | 16 | 0.00292 | Atg9b,Dnm1l,Mfn2,Nedd4,Npc1,Pik3c3,Pik3ca,Pik3r4,Rab3gap1,Rock1,Sh3glb1,Snx14,Usp33,Vps36, Vps4a,Vta1 |
| GO.0048585 | negative regulation of response to stimulus | 9 | 0.00314 | Map3k7,Mfn2,Mtor,Nedd4,Npc1,Rb1cc1,Sh3glb1,Sirt1,Snx14 |
| GO.0019222 | regulation of metabolic process | 19 | 0.0033 | Csnk2a1,Dnm1l,Eif2ak4,Larp1,Map3k7,Npc1,Nrbf2,Pafah1b2,Pik3ca,Pik3r4,Rb1cc1,Sh3glb1,Sirt1,Stom, Tbk1,Ubxn2b,Ulk2,Usp33,Vps36 |
| GO.0051302 | regulation of cell division | 5 | 0.0033 | Csnk2a1,Pik3c3,Pik3r4,Sh3glb1,Vps4a |
| GO.1902580 | single-organism cellular localization | 7 | 0.00349 | Mfn2,Nedd4,Npc1,Pik3r4,Rab3gap1,Sh3glb1,Vps4a |
| GO.0016032 | viral process | 4 | 0.00351 | Nedd4,Npc1,Vps4a,Vta1 |
| GO.0044700 | single organism signaling | 15 | 0.00357 | Csnk2a1,Dnm1l,Eif2ak4,Larp1,Map3k7,Mtor,Nedd4,Npc1,Pik3c3,Pik3ca,Rab3gap1,Rb1cc1,Rock1,Sirt1, Ulk2 |
| GO.0043549 | regulation of kinase activity | 7 | 0.0038 | Map3k7,Mtor,Nrbf2,Pik3ca,Pik3r4,Sh3glb1,Sirt1 |
| GO.1902531 | regulation of intracellular signal transduction | 9 | 0.00389 | Dnm1l,Map3k7,Mfn2,Mtor,Nedd4,Rab3gap1,Sh3glb1,Sirt1,Tbk1 |
| GO.0034198 | cellular response to amino acid starvation | 2 | 0.00431 | Eif2ak4,Sh3glb1 |
| GO.0034613 | cellular protein localization | 8 | 0.00436 | Atg9b,Dnm1l,Mfn2,Nedd4,Npc1,Pik3r4,Rab3gap1,Sh3glb1 |
| GO.0051247 | positive regulation of protein metabolic process | 9 | 0.00436 | Csnk2a1,Larp1,Map3k7,Mtor,Nedd4,Pik3ca,Rb1cc1,Sirt1,Tbk1 |
| GO.0009893 | positive regulation of metabolic process | 14 | 0.00521 | Csnk2a1,Eif2ak4,Larp1,Map3k7,Mtor,Nedd4,Pafah1b2,Pik3ca,Pik3r4,Rb1cc1,Sh3glb1,Sirt1,Stom,Tbk1 |
| GO.0043550 | regulation of lipid kinase activity | 3 | 0.00559 | Nrbf2,Pik3r4,Sh3glb1 |
| GO.0023056 | positive regulation of signaling | 9 | 0.00592 | Csnk2a1,Dnm1l,Map3k7,Mtor,Nedd4,Rab3gap1,Sh3glb1,Sirt1,Tbk1 |
| GO.0043254 | regulation of protein complex assembly | 5 | 0.00662 | Dnm1l,Map3k7,Mtor,Rab3gap1,Sh3glb1 |
| GO.0044763 | single-organism cellular process | 24 | 0.00662 | Atg9b,Atp6v1h,Csnk2a1,Dnm1l,Eif2ak4,Larp1,Map1lc3a,Map3k7,Mfn2,Mtor,Pafah1b2,Pik3c3,Pik3ca, Pik3r4,Rab3gap1,Rb1cc1,Rock1,Sh3glb1,Sirt1,Tbk1,Ubxn2b,Ulk2,Usp33,Vps4a |
| GO.0007165 | signal transduction | 14 | 0.00708 | Csnk2a1,Dnm1l,Eif2ak4,Larp1,Map3k7,Mtor,Nedd4,Npc1,Pik3c3,Pik3ca,Rb1cc1,Rock1,Sirt1,Ulk2 |
| GO.0010941 | regulation of cell death | 9 | 0.00708 | Csnk2a1,Dnm1l,Map3k7,Npc1,Pik3ca,Rb1cc1,Rock1,Sh3glb1,Sirt1 |
| GO.0044238 | primary metabolic process | 21 | 0.00708 | Csnk2a1,Eif2ak4,Larp1,Map3k7,Mtor,Npc1,Nrbf2,Pafah1b2,Pik3ca,Pik3r4,Rb1cc1,Rock1,Sh3glb1,Sirt1, Tbk1,U2af1,Ubxn2b,Ulk2,Usp33,Vps36,Vps4a |
| GO.2001244 | positive regulation of intrinsic apoptotic signaling pathway | 3 | 0.00753 | Dnm1l,Sh3glb1,Sirt1 |
| GO.0043066 | negative regulation of apoptotic process | 7 | 0.00765 | Csnk2a1,Map3k7,Pik3ca,Rb1cc1,Rock1,Sh3glb1,Sirt1 |
| GO.1902533 | positive regulation of intracellular signal transduction | 7 | 0.008 | Dnm1l,Map3k7,Mtor,Nedd4,Sh3glb1,Sirt1,Tbk1 |
| GO.0042327 | positive regulation of phosphorylation | 7 | 0.00823 | Map3k7,Mtor,Pik3ca,Pik3r4,Rb1cc1,Sh3glb1,Sirt1 |
| GO.0007005 | mitochondrion organization | 5 | 0.00862 | Atg9b,Dnm1l,Map1lc3a,Mfn2,Sh3glb1 |
| GO.0034497 | protein localization to pre-autophagosomal structure | 2 | 0.00864 | Atg9b,Mfn2 |
| GO.0071704 | organic substance metabolic process | 21 | 0.0105 | Csnk2a1,Eif2ak4,Larp1,Map3k7,Mtor,Npc1,Nrbf2,Pafah1b2,Pik3ca,Pik3r4,Rb1cc1,Rock1,Sh3glb1,Sirt1, Tbk1,U2af1,Ubxn2b,Ulk2,Usp33,Vps36,Vps4a |
| GO.0051050 | positive regulation of transport | 7 | 0.0107 | Dnm1l,Nedd4,Rab3gap1,Sh3glb1,Sirt1,Stom,Vps4a |
| GO.0019220 | regulation of phosphate metabolic process | 9 | 0.0108 | Dnm1l,Map3k7,Mtor,Nrbf2,Pik3ca,Pik3r4,Rb1cc1,Sh3glb1,Sirt1 |
| GO.1902589 | single-organism organelle organization | 10 | 0.0111 | Atg9b,Dnm1l,Map1lc3a,Mfn2,Pik3c3,Pik3r4,Rock1,Sirt1,Usp33,Vps4a |
| GO.0031647 | regulation of protein stability | 4 | 0.012 | Rab3gap1,Sh3glb1,Sirt1,Usp33 |
| GO.0016242 | negative regulation of macroautophagy | 2 | 0.0125 | Mtor,Npc1 |
| GO.0032880 | regulation of protein localization | 7 | 0.0125 | Dnm1l,Mtor,Pik3c3,Sh3glb1,Sirt1,Stom,Vps4a |
| GO.1902582 | single-organism intracellular transport | 7 | 0.0135 | Mfn2,Nedd4,Npc1,Pik3c3,Pik3r4,Vps36,Vps4a |
| GO.0016197 | endosomal transport | 4 | 0.0136 | Pik3c3,Pik3r4,Vps36,Vps4a |
| GO.0007034 | vacuolar transport | 3 | 0.0139 | Nedd4,Npc1,Pik3r4 |
| GO.0009967 | positive regulation of signal transduction | 8 | 0.0139 | Csnk2a1,Dnm1l,Map3k7,Mtor,Nedd4,Sh3glb1,Sirt1,Tbk1 |
| GO.0036092 | phosphatidylinositol-3-phosphate biosynthetic process | 2 | 0.0139 | Pik3c3,Pik3ca |
| GO.0046755 | viral budding | 2 | 0.0139 | Vps4a,Vta1 |
| GO.1902254 | negative regulation of intrinsic apoptotic signaling pathway by p53 class mediator | 2 | 0.0139 | Sh3glb1,Sirt1 |
| GO.0006915 | apoptotic process | 7 | 0.0143 | Csnk2a1,Dnm1l,Map3k7,Mfn2,Rock1,Sh3glb1,Sirt1 |
| GO.0051246 | regulation of protein metabolic process | 11 | 0.0149 | Csnk2a1,Eif2ak4,Larp1,Map3k7,Mtor,Nedd4,Pik3ca,Rb1cc1,Sirt1,Tbk1,Usp33 |
| GO.0048522 | positive regulation of cellular process | 15 | 0.016 | Csnk2a1,Dnm1l,Larp1,Map3k7,Nedd4,Pafah1b2,Pik3ca,Pik3r4,Rab3gap1,Rb1cc1,Rock1,Sh3glb1,Sirt1, Stom,Vps4a |
| GO.0019058 | viral life cycle | 3 | 0.0165 | Npc1,Vps4a,Vta1 |
| GO.2001233 | regulation of apoptotic signaling pathway | 5 | 0.0165 | Dnm1l,Map3k7,Rb1cc1,Sh3glb1,Sirt1 |
| GO.0042325 | regulation of phosphorylation | 8 | 0.0166 | Map3k7,Mtor,Nrbf2,Pik3ca,Pik3r4,Rb1cc1,Sh3glb1,Sirt1 |
| GO.0051704 | multi-organism process | 9 | 0.0166 | Mtor,Nedd4,Npc1,Pafah1b2,Sirt1,Stom,Tbk1,Vps4a,Vta1 |
| GO.0072594 | establishment of protein localization to organelle | 4 | 0.0166 | Mfn2,Nedd4,Pik3r4,Rab3gap1 |
| GO.0044087 | regulation of cellular component biogenesis | 6 | 0.0181 | Dnm1l,Map3k7,Mtor,Rab3gap1,Sh3glb1,Vps4a |
| GO.2001234 | negative regulation of apoptotic signaling pathway | 4 | 0.0181 | Map3k7,Rb1cc1,Sh3glb1,Sirt1 |
| GO.0009968 | negative regulation of signal transduction | 7 | 0.0188 | Map3k7,Mfn2,Nedd4,Rb1cc1,Sh3glb1,Sirt1,Snx14 |
| GO.0033674 | positive regulation of kinase activity | 5 | 0.019 | Map3k7,Pik3ca,Pik3r4,Sh3glb1,Sirt1 |
| GO.0009057 | macromolecule catabolic process | 6 | 0.0191 | Pik3r4,Sh3glb1,Sirt1,Ubxn2b,Usp33,Vps4a |
| GO.0031929 | TOR signaling | 2 | 0.0191 | Larp1,Mtor |
| GO.0042981 | regulation of apoptotic process | 8 | 0.0191 | Csnk2a1,Dnm1l,Map3k7,Pik3ca,Rb1cc1,Rock1,Sh3glb1,Sirt1 |
| GO.0043162 | ubiquitin-dependent protein catabolic process via the multivesicular body sorting pathway | 2 | 0.0191 | Nedd4,Vps4a |
| GO.0035556 | intracellular signal transduction | 8 | 0.0219 | Larp1,Map3k7,Mtor,Pik3c3,Pik3ca,Rb1cc1,Rock1,Sirt1 |
| GO.0051223 | regulation of protein transport | 6 | 0.0219 | Dnm1l,Mtor,Pik3c3,Sh3glb1,Sirt1,Stom |
| GO.0046486 | glycerolipid metabolic process | 4 | 0.0234 | Pik3c3,Pik3ca,Sh3glb1,Sirt1 |
| GO.0010564 | regulation of cell cycle process | 5 | 0.0269 | Csnk2a1,Pik3c3,Pik3r4,Sh3glb1,Vps4a |
| GO.0009653 | anatomical structure morphogenesis | 10 | 0.0274 | Dnm1l,Map3k7,Mfn2,Nedd4,Pik3ca,Rab3gap1,Rock1,Sirt1,Ulk2,Usp33 |
| GO.0010604 | positive regulation of macromolecule metabolic process | 11 | 0.0274 | Csnk2a1,Eif2ak4,Larp1,Map3k7,Mtor,Nedd4,Pik3ca,Rab3gap1,Rb1cc1,Sirt1,Tbk1 |
| GO.0015031 | protein transport | 7 | 0.0285 | Mfn2,Nedd4,Pik3r4,Snx14,Vps36,Vps4a,Vta1 |
| GO.0046474 | glycerophospholipid biosynthetic process | 3 | 0.0308 | Pik3c3,Pik3ca,Sh3glb1 |
| GO.0051649 | establishment of localization in cell | 8 | 0.0308 | Mfn2,Nedd4,Npc1,Pik3c3,Pik3r4,Rab3gap1,Vps36,Vps4a |
| GO.0019068 | virion assembly | 2 | 0.031 | Vps4a, Vta1 |
| GO.1903827 | regulation of cellular protein localization | 5 | 0.031 | Mtor,Sh3glb1,Sirt1,Stom,Vps4a |
| GO.0006810 | transport | 12 | 0.0312 | Dnm1l,Mfn2,Nedd4,Npc1,Pik3c3,Pik3r4,Rab3gap1,Snx14,Usp33,Vps36,Vps4a,Vta1 |
| GO.0010822 | positive regulation of mitochondrion organization | 4 | 0.0312 | Dnm1l,Pik3ca,Sh3glb1,Stom |
| GO.0048519 | negative regulation of biological process | 14 | 0.0314 | Csnk2a1,Eif2ak4,Map3k7,Mfn2,Mtor,Pik3ca,Rb1cc1,Rock1,Sh3glb1,Sirt1,Snx14,Tbk1,Ulk2,Vps4a |
| GO.1902578 | single-organism localization | 11 | 0.0314 | Atp6v1h,Mfn2,Nedd4,Npc1,Pik3c3,Pik3r4,Rab3gap1,Sh3glb1,Vps36,Vps4a,Vta1 |
| GO.0006623 | protein targeting to vacuole | 2 | 0.0328 | Nedd4,Pik3r4 |
| GO.0044804 | nucleophagy | 2 | 0.0328 | Atg9b,Map1lc3a |
| GO.0019216 | regulation of lipid metabolic process | 4 | 0.0346 | Nrbf2,Pik3r4,Sh3glb1,Sirt1 |
| GO.1903533 | regulation of protein targeting | 4 | 0.0387 | Mtor,Sh3glb1,Sirt1,Stom |
| GO.0031325 | positive regulation of cellular metabolic process | 11 | 0.0413 | Larp1,Map3k7,Mtor,Pafah1b2,Pik3ca,Pik3r4,Rb1cc1,Sh3glb1,Sirt1,Stom,Tbk1 |
| GO.0032386 | regulation of intracellular transport | 5 | 0.0433 | Mtor,Nedd4,Sh3glb1,Sirt1,Stom |
| GO.0045834 | positive regulation of lipid metabolic process | 3 | 0.0438 | Mtor,Pik3r4,Sh3glb1 |
| GO.0031330 | negative regulation of cellular catabolic process | 3 | 0.0471 | Csnk2a1,Mtor,Npc1 |
| GO.0015991 | ATP hydrolysis coupled proton transport | 2 | 0.0472 | Atp6v1c1,Atp6v1h |
| GO.0048523 | negative regulation of cellular process | 13 | 0.0497 | Csnk2a1,Eif2ak4,Map3k7,Mfn2,Mtor,Pik3ca,Rb1cc1,Rock1,Sh3glb1,Sirt1,Snx14,Ulk2,Vps4a |
