# Supplementary material for: Evolution of mammalian longevity: age-related increase in autophagy in bats compared to other mammals
Source: Aging (Albany NY). 2021 Mar 21;13(6):7998–8025. doi: 10.18632/aging.202852 (PMC8034928; doi:10.18632/aging.202852)
Supplement: Supplementary Tables 5, 6, and 7 [file aging-13-202852-s007.pdf]

Supplementary Table 5. Functional enrichment in KEGG pathways of the STRING protein-protein interaction network including genes under selective pressure in bat lineages, genes showing bat-specific upregulation with age, and LC3.

Pathway ID	Pathway description	Observed gene count	FDR	Matching protein in the network
5169	Epstein-Barr virus infection	6	0.000128	Csnk2a1,Eif2ak4,Map3k7,Nedd4,Pik3ca,Tbk1
5162	Measles	5	0.000257	Csnk2a1,Eif2ak4,Map3k7,Pik3ca,Tbk1
4144	Endocytosis	5	0.00138	Nedd4,Sh3glb1,Vps36,Vps4a,Vta1
4140	Regulation of autophagy	3	0.00208	Pik3c3,Pik3r4,Ulk2
4152	AMPK signaling pathway	4	0.00211	Map3k7,Mtor,Pik3ca,Sirt1
4150	mTOR signaling pathway	3	0.00495	Mtor,Pik3ca,Ulk2
5168	Herpes simplex infection	4	0.00832	Csnk2a1,Eif2ak4,Map3k7,Tbk1
4620	Toll-like receptor signaling pathway	3	0.016	Map3k7,Pik3ca,Tbk1
4668	TNF signaling pathway	3	0.0178	Dnm1l,Map3k7,Pik3ca
5160	Hepatitis C	3	0.03	Eif2ak4,Pik3ca,Tbk1
5206	MicroRNAs in cancer	3	0.0303	Mtor,Rock1,Sirt1
4145	Phagosome	3	0.0424	Atp6v1c1,Atp6v1h,Pik3c3
5164	Influenza A	3	0.0442	Eif2ak4,Pik3ca,Tbk1
4930	Type II diabetes mellitus	2	0.0498	Mtor,Pik3ca

Supplementary Table 6. Genes enriched in the parental GO term 'autophagy' that showed correlation with age in M.

Gene	Bat	Human	Mouse
PIK3CA	0.483	-0.233	-0.568
ROCK1	0.468	-0.091	-0.41
RB1CC1	0.446	-0.143	-0.164
ATP6V1E2	0.438	-0.469	NA
RAB12	0.423	0.075	0.254
CSNK2A1	0.415	-0.015	-0.406
SCFD1	0.385	-0.235	0.027
NEDD4	0.383	-0.043	-0.1
UVRAG	0.36	0.156	0.087
POLR3A	0.343	0.17	-0.185
ATG3	0.341	-0.117	0.508
MTDH	0.318	-0.148	0.586
CISD2	0.314	0.079	0.28
PIK3CB	0.308	-0.14	0.497
ATG4C	0.296	-0.281	0.656
PAFAH1B2	0.293	-0.278	-0.097
MAPK8	0.286	0.099	-0.187
USP33	0.278	-0.237	-0.165
STAM	0.276	0.029	-0.213
FIS1	0.275	0.111	-0.032
MID2	0.274	0.218	0.262
TRAPPC8	0.274	-0.167	-0.066
ULK2	0.271	-0.017	-0.312
FBXW7	0.267	0.074	0.112
TOMM70	0.267	NA	NA
SIRT1	0.243	-0.091	-0.566
HERC1	0.241	0.06	0.086
VPS26A	0.24	0.046	0.367
PRKAB2	0.234	-0.157	0.551
STAM2	0.226	-0.299	0.334
SNX14	0.214	-0.27	-0.158
EP300	0.213	-0.17	0.013
VTA1	0.208	-0.302	-0.327
MAP3K7	0.203	-0.213	-0.247
TBC1D5	0.198	-0.231	0.111
TBK1	0.198	-0.218	-0.215
SNX5	0.19	0.143	-0.653
VPS36	0.179	-0.207	-0.483
NRBF2	0.177	-0.236	-0.361
WDFY3	0.176	-0.134	0.703
DNM1L	0.168	-0.081	-0.245
VTI1A	0.164	0.042	0.482
UBXN2B	0.146	-0.14	-0.179
EIF2AK4	0.141	-0.069	-0.361
WDR41	0.138	-0.121	0.275
RIPK2	0.137	0.232	-0.214

SH3GLB1	0.132	-0.149	-0.039
ATG4A	0.121	-0.063	0.094
PIK3R4	0.11	-0.227	-0.457
ATG5	0.108	-0.082	0.227
IFT88	0.104	-0.124	0.268
VIPAS39	0.103	-0.202	NA
ATP6V1H	0.099	-0.172	-0.032
SPTLC1	0.093	-0.2	0.072
PIK3C3	0.077	-0.268	-0.269
ATG10	0.076	-0.105	0.147
PTK2	0.061	0.109	0.484
RAB3GAP1	0.053	-0.013	-0.567
AMBRA1	0.052	0.153	-0.323
EPM2A	0.048	0.025	0.268
ANXA7	0.046	-0.2	0.383
ATP6V1C1	0.03	-0.186	-0.09
U2AF1	0.03	-0.053	-0.207
ATP6V0A1	0.021	0.015	0.181
RHEB	-0.006	-0.018	0.356
VPS4B	-0.023	-0.248	0.202
ACTL6A	-0.066	-0.327	-0.318
SREBF2	-0.074	-0.037	-0.03
TPCN1	-0.08	-0.151	0.112
MTMR3	-0.084	-0.012	0.067

myotis bats. The Spearman's rank correlation coefficients between their expression and age in bat, human and mouse were extracted from Huang et al. (2019). NA – transcript not detected in the blood transcriptome.

Supplementary Table 7. Taxonomic information: List of species included in the study, their record lifespan from AnAge, source of the samples, age ranges of individuals included in the study and sampling permit details.

Species	Max. lifespan	Source	Sample	Age range	Permit details for sampling wild bat populations
M. myotis	37.1yrs	Wild population, Brittany, France	wing punches	0 - 7+yrs	Arrêté préfectoral (18/07/2013 and 05/08/2013) issued to Eric Petit, Frédéric Touzalin and Sébastien Puechmaille by Préfet du Morbihan, valid between 15th June - 15th September; 2013-2017 inclusive
P. kuhlii	8yrs	Wild population, Krašnja, Slovenia<br>Wild population, Piemonte, Italy	wing punches	0 – 7+ yrs	Aut. Prot. N°: 13040, issued to Roberto Toffoli and Andrea Locatelli by the Italian ministry for the territory and sea safeguard (Ministero della tutela del territorio e del mare)<br>Aut N°: 35601-35/2010-6, issued to Primož Presetnik by the Slovenian Environment Agency (Agencija Republike Slovenije za okolje)
M. musculus (C57BL/6J strain)	4 yrs	Conboy laboratory (Department of Bioengineering, Berkeley Research, University of California) and O'Neill laboratory (School of Biochemistry and Immunology, Trinity College Dublin)	ear clippings	2 – 22 months	not applicable
M. musculus (NHEJ background)	na	Gorbanova laboratory (Departments of Biology and Medicine, University of Rochester, Rochester, NY 14627, USA)	flank-skin derived primary fibroblasts developed as decribed in Vaidya et al. (2014)	3 and 28 months	not applicable
