# Supplementary material for: Evolution of mammalian longevity: age-related increase in autophagy in bats compared to other mammals
Source: Aging (Albany NY). 2021 Mar 21;13(6):7998–8025. doi: 10.18632/aging.202852 (PMC8034928; doi:10.18632/aging.202852)
Supplement: Supplementary Table 8 [file aging-13-202852-s008.docx]

Supplementary Table 8. List of genes involved in mammalian autophagy retrived from AmiGO database using search term 'autophagy'. The redundant names representing the same gene and genes of unknown function were removed.

| **Gene** | | | | | |
| --- | --- | --- | --- | --- | --- |
| *5HT2B* | *Csnk2a2* | *Kat2a* | *Pi4k2a* | *SNF8* | *Usp30* |
| *Abl1* | *Cspg5* | *Kat8* | *Pik3c2a* | *SNRNP70* | *Usp33* |
| *Abl2* | *Ctsa* | *Kcnq1* | *Pik3c2b* | *Snrpb* | *Usp36* |
| *Acbd5* | *Ctsb* | *Kdr* | *Pik3c3* | *Snrpb2* | *Uvrag* |
| *Acin1* | *Ctsd* | *KIAA1324* | *Pik3ca* | *Snrpd1* | *Vamp7* |
| *Ackr3* | *Ctsk* | *KIAA1549L* | *Pik3cb* | *Snrpf* | *Vamp8* |
| *Actl6a* | *Cttn* | *Krcc1* | *Pik3r2* | *Sntg1* | *Vcp* |
| *Adamts7* | *Dap* | *Krt15* | *Pik3r4* | *Snx14* | *Vdac1* |
| *Adrb2* | *DAPK1* | *Lamp1* | *Pikfyve* | *SNX5* | *Vipas39* |
| *Ager* | *DAPK2* | *Lamp2* | *Pim2* | *SNX6* | *Vmp1* |
| *Akr1e2* | *DAPK3* | *LAMTOR1* | *Pink1* | *Soga1* | *Vps11* |
| *Akt1* | *Dapl1* | *LAMTOR2* | *Pip4k2a* | *Soga3* | *Vps13a* |
| *Alkbh5* | *Dcn* | *LAMTOR3* | *Pip4k2b* | *Spata18* | *Vps13c* |
| *Alpk1* | *Dear1* | *LAMTOR4* | *Pip4k2c* | *Sptlc1* | *Vps16* |
| *Ambra1* | *Depdc5* | *LAMTOR5* | *Plekhm1* | *Sptlc2* | *Vps18* |
| *Anxa5* | *Dkkl1* | *Larp1* | *Plk2* | *Sqstm1* | *VPS25* |
| *Anxa7* | *Dnaaf2* | *Larp1b* | *Plod2* | *Srebf1* | *VPS26A* |
| *Arsa* | *Dnm1l* | *Leng9* | *Pnpo* | *Srebf2* | *VPS26B* |
| *Arsb* | *Dpf3* | *Lep* | *Poldip2* | *Srpx* | *VPS28* |
| *Asb2* | *Dram1* | *Lepr* | *Polr3a* | *STAM* | *Vps33a* |
| *Atg10* | *Dram2* | *Lgals8* | *Ppargc1a* | *STAM2* | *Vps33b* |
| *Atg101* | *DYNLL1* | *Lix1* | *Prkaa1* | *Stat2* | *VPS35* |
| *Atg12* | *DYNLL2* | *Lix1l* | *Prkaa2* | *Stbd1* | *VPS36* |
| *Atg13* | *DZANK1* | *Lmcd1* | *PRKAB1* | *Stk11* | *VPS37A* |
| *Atg14* | *EEF1A1* | *Lmx1b* | *PRKAB2* | *Stom* | *VPS37B* |
| *Atg16l1* | *EEF1A2* | *Lrrk2* | *PRKAG1* | *Stx12* | *VPS37C* |
| *Atg16l2* | *Ehmt2* | *Lrsam1* | *PRKAG2* | *Stx17* | *VPS37D* |
| *Atg2a* | *Ei24* | *Lsm4* | *PRKAG3* | *SUPT20H* | *Vps39* |
| *Atg2b* | *Eif2ak4* | *Lzts1* | *Prkd2* | *Supt3h* | *Vps41* |
| *Atg3* | *Eif2s1* | *Map1a* | *Prkg1* | *Supt5* | *Vps4a* |
| *Atg4a* | *EP300* | *Map1lc3a* | *PSAP* | *Svip* | *Vps4b* |
| *Atg4b* | *Epg5* | *MAP1LC3B* | *Psen1* | *TAB2* | *Vps51* |
| *Atg4c* | *Epm2a* | *MAP1S* | *Pycard* | *TAB3* | *VTA1* |
| *Atg4d* | *ERN1* | *Map2k1* | *Qsox1* | *TBC1D12* | *Vti1a* |
| *Atg5* | *Eva1a* | *Map3k12* | *Rab12* | *Tbc1d14* | *Vti1b* |
| *Atg7* | *Eva1b* | *Mapk3* | *Rab1a* | *Tbc1d17* | *Wac* |
| *Atg9a* | *EXOC1* | *MAPK8* | *Rab1b* | *Tbc1d25* | *Wash1* |
| *Atg9b* | *EXOC4* | *Mapt* | *Rab20* | *Tbc1d5* | *Wbp11* |
| *Atm* | *EXOC7* | *MARK2* | *Rab23* | *Tbk1* | *Wdfy3* |
| *Atp13a2* | *EXOC8* | *Mbd5* | *Rab24* | *Tcirg1* | *Wdr24* |
| *Atp1b1* | *Fam131b* | *Mcl1* | *Rab33a* | *Tead4* | *Wdr45* |
| *Atp6v0a1* | *Fam13b* | *Mcoln1* | *Rab33b* | *Tecpr1* | *Wdr45b* |
| *ATP6V0A2* | *Fancc* | *Mdh1* | *RAB39A* | *TECPR2* | *Wdr6* |
| *ATP6V0B* | *Fancf* | *Mefv* | *Rab3gap1* | *Tfeb* | *Wdr75* |
| *ATP6V0C* | *Fancl* | *MET* | *Rab3gap2* | *Ticam1* | *Wipi1* |
| *ATP6V0D1* | *Fbxl2* | *Mex3c* | *RAB5A* | *Tigar* | *Wipi2* |
| *ATP6V0E1* | *Fbxo7* | *MFN1* | *Rab7a* | *TLK2* | *Xbp1* |
| *ATP6V0E2* | *Fbxw7* | *Mfn2* | *Ralb* | *Tlr2* | *Yipf1* |
| *ATP6V1A* | *Fez1* | *Mfsd8* | *Rasip1* | *Tlr9* | *Zbtb17* |
| *ATP6V1B2* | *Fez2* | *Mid2* | *Rb1cc1* | *Tm9sf1* | *Zc3h12a* |
| *ATP6V1C1* | *Fgf14* | *MLST8* | *Rbm18* | *Tmbim6* | *Zcchc17* |
| *ATP6V1C2* | *Fgf7* | *Mrps10* | *Reep2* | *Tmem208* | *Zdhhc8* |
| *ATP6V1D* | *Fis1* | *Mrps2* | *Rep15* | *Tmem39a* | *Zfyve1* |
| *ATP6V1E1* | *Flcn* | *Mstn* | *Rfwd3* | *Tmem39b* | *Zkscan3* |
| *ATP6V1E2* | *Fnbp1l* | *MT-III* | *RGD1305347* | *Tmem59* | *Zkscan4* |
| *ATP6V1G1* | *Foxo1* | *Mtcl1* | *RGD1310209* | *Tmem74* | *ZNF189* |
| *ATP6V1G2* | *Fundc1* | *Mtdh* | *RGD1565641* | *Tnfaip3* | *ZNF593* |
| *ATP6V1H* | *Fundc2* | *MTERF3* | *RGS19* | *Tnik* |  |
| *BAD* | *Fxr2* | *Mtm1* | *RHEB* | *Tollip* |  |
| *Bcl2* | *Fyco1* | *MTMR14* | *Rims3* | *TOMM20* |  |
| *Becn1* | *Fzd5* | *Mtmr3* | *Ripk2* | *TOMM22* |  |
| *Becn2* | *Gabarap* | *Mtor* | *Rnf152* | *TOMM40* |  |
| *Bloc1s1* | *Gabarapl1* | *Mtss1* | *Rnf185* | *TOMM5* |  |
| *Bnip1* | *GAPDH* | *MVB12A* | *Rnf41* | *TOMM6* |  |
| *Bnip3* | *Gata4* | *MYH11* | *Rnf5* | *Tomm7* |  |
| *Bnip3l* | *Gba* | *Mylk* | *Rpl28* | *TOMM70* |  |
| *Boc* | *GBRL2* | *Myom1* | *RPS27A* | *Tp53* |  |
| *Bok* | *Gfap* | *Nbr1* | *RPTOR* | *Tp53inp1* |  |
| *C19orf12* | *Gmip* | *Ndufb9* | *Rraga* | *Tp53inp2* |  |
| *C1orf210* | *Golga2* | *NEDD4* | *Rragb* | *Tpcn1* |  |
| *C6orf106* | *Gopc* | *Nefm* | *Rragc* | *Tpcn2* |  |
| *C8orf59* | *gpr81* | *Nhlrc1* | *Rragd* | *TPPC8* |  |
| *C9orf72* | *Gsk3a* | *Nme2* | *Rubcn* | *Trim13* |  |
| *CA7* | *Hapln1* | *Nod1* | *Ruvbl1* | *Trim17* |  |
| *Calcoco2* | *HAX1* | *Nod2* | *S100a8* | *TRIM22* |  |
| *CAPN1* | *Hdac6* | *Npc1* | *S100a9* | *Trim5* |  |
| *Capn10* | *Herc1* | *Nprl2* | *Scfd1* | *Trim61* |  |
| *CAPNS1* | *HGF* | *Nprl3* | *Scoc* | *Trim65* |  |
| *CAPS* | *HGS* | *Nr2c2* | *Serpinb10* | *Trim8* |  |
| *Casp1* | *Hif1a* | *Nrbf2* | *Sesn2* | *Trp53inp1* |  |
| *CASP3* | *Hilpda* | *Nrbp2* | *Sfrp4* | *Tsc1* |  |
| *Cd93* | *HIST1H3E* | *Nthl1* | *Sh3bp4* | *Tsc2* |  |
| *Cdc37* | *Hk2* | *Nup93* | *Sh3glb1* | *TSG101* |  |
| *CDK5* | *Hmgb1* | *Obscn* | *Sirt1* | *Tspo* |  |
| *CDK5R1* | *Hmox1* | *Optn* | *Sirt2* | *Txlna* |  |
| *Chaf1b* | *Hsf2bp* | *OSBPL7* | *Slc17a9* | *U2af1* |  |
| *CHMP2A* | *Hsp90aa1* | *P2rx5* | *Slc1a3* | *U2af2* |  |
| *CHMP2B* | *Hsp90ab1* | *Pacs2* | *Slc1a4* | *Uba52* |  |
| *CHMP4A* | *Hspa8* | *Pafah1b2* | *Slc22a3* | *UBB* |  |
| *Chmp4b* | *HSPB1* | *Park2* | *Slc25a19* | *Ubqln1* |  |
| *CHMP4C* | *HTRA2* | *Park7* | *Slc35b3* | *Ubqln2* |  |
| *CHMP6* | *Htt* | *Parl* | *Slc35c1* | *Ubqln4* |  |
| *Chst3* | *Ift20* | *Pdk1* | *Slc37a4* | *UCHL1* |  |
| *Cisd2* | *Ift88* | *Pdk4* | *Slc6a1* | *Ulk1* |  |
| *Cldn7* | *Ippk* | *Pex13* | *Smurf1* | *Ulk2* |  |
| *Clec16a* | *Ist1* | *Pex3* | *Snap29* | *Ulk3* |  |
| *Cln3* | *Itgb4* | *Pfkp* | *Snca* | *Usp10* |  |
| *Clvs1* | *Itpkc* | *Phf23* |  | *Usp13* |  |
| *Cox8a* | *ITPR1* | *Phyhip* |  |  |  |
| *CRSP1* |  |  |  |  |  |
