# Supplementary material for: Evolution of mammalian longevity: age-related increase in autophagy in bats compared to other mammals
Source: Aging (Albany NY). 2021 Mar 21;13(6):7998–8025. doi: 10.18632/aging.202852 (PMC8034928; doi:10.18632/aging.202852)
Supplement: Supplementary Table 9 [file aging-13-202852-s009.pdf]

Supplementary Table 9. RefSeq mammals used.

Mammalian genome	Number of genes	Order
Loxodonta africana	244	Afrotheria
Elephantulus edwardii	232	Afrotheria
Echinops telfairi	254	Afrotheria
Orycteropus afer	262	Afrotheria
Dasypus novemcinctus	241	Xenarthra
Lipotes vexillifer	249	Cetartiodactyla
Orcinus orca	261	Cetartiodactyla
Tursiops truncatus	168	Cetartiodactyla
Physeter catodon	156	Cetartiodactyla
Balaenoptera acutorostrata	240	Cetartiodactyla
Bison bison	254	Cetartiodactyla
Bos Taurus	261	Cetartiodactyla
Ovis aries	213	Cetartiodactyla
Sus scrofa	228	Cetartiodactyla
Camelus bactrianus	252	Cetartiodactyla
Vicugna pacos	248	Cetartiodactyla
Equus caballus	249	Perissodactyla
Manis javanica	248	Pholidota
Felis catus	263	Carnivora
Panthera tigris	238	Carnivora
Ailuropoda melanoleuca	255	Carnivora
Mustela putorius	61	Carnivora
Odobenus rosmarus	247	Carnivora
Canis lupus	134	Carnivora
Pteropus alecto	250	Chiroptera
Pteropus vampyrus	258	Chiroptera
Rousettus aegyptiacus	250	Chiroptera
Rhinolophus sinicus	263	Chiroptera
Hipposideros armiger	240	Chiroptera
Miniopterus natalensis	256	Chiroptera
Eptesicus fuscus	261	Chiroptera
Myotis brandtii	257	Chiroptera
Myotis lucifugus	244	Chiroptera
Myotis davidii	254	Chiroptera
Erinaceus europaeus	252	Eulipotyphla
Sorex araneus	121	Eulipotyphla
Galeopterus variegatus	69	Dermoptera
Otolemur garnettii	260	Primates
Microcebus murinus	249	Primates
Callithrix jacchus	235	Primates
Colobus angolensis	211	Primates
Papio anubis	171	Primates
Mandrillus leucophaeus	236	Primates

Macaca mulatta	259	Primates
Pongo abelii	236	Primates
Gorilla gorilla	235	Primates
Homo sapiens	267	Primates
Pan paniscus	254	Primates
Pan troglodytes	246	Primates
Tupaia chinensis	247	Scandentia
Oryctolagus cuniculus	237	Lagomorpha
Microtus ochrogaster	260	Rodentia
Mesocricetus auratus	255	Rodentia
Rattus norvegicus	263	Rodentia
Mus musculus	264	Rodentia
Jaculus jaculus	261	Rodentia
Dipodomys ordii	264	Rodentia
Ictidomys tridecemlineatus	262	Rodentia
Chinchilla lanigera	258	Rodentia
Octodon degus	137	Rodentia
Cavia porcellus	255	Rodentia
Heterocephalus glaber	253	Rodentia

The mammals for which a RefSeq annotation was available, the number of autophagy-associated genes found and mammalian order is displayed.
