# Supplementary material for: Evolution of mammalian longevity: age-related increase in autophagy in bats compared to other mammals
Source: Aging (Albany NY). 2021 Mar 21;13(6):7998–8025. doi: 10.18632/aging.202852 (PMC8034928; doi:10.18632/aging.202852)
Supplement: Supplementary Table 10 [file aging-13-202852-s010.docx]

Supplementary Table 10. Autophagy-associated genes tested for selection pressure across eutherian mammals.

| **Gene** | **Number of taxa with CDS available** | |
| --- | --- | --- |
|  | **RefSeq** | **RefSeq + MAKER** |
| *ABL2* | 55 | 59 |
| *ACKR3* | 57 | 62 |
| *ACTL6A* | 58 | 62 |
| *ALKBH5* | 55 | 59 |
| *ANXA5* | 55 | 60 |
| *ARSA* | 59 | 63 |
| *ARSB* | 52 | 56 |
| *ASB2* | 44 | 47 |
| *ATG101* | 55 | 62 |
| *ATG12* | 53 | 57 |
| *ATG13* | 59 | 66 |
| *ATG16L1* | 56 | 61 |
| *ATG3* | 47 | 52 |
| *ATG4A* | 57 | 62 |
| *ATG4B* | 57 | 60 |
| *ATG4C* | 57 | 62 |
| *ATG4D* | 56 | 63 |
| *ATG5* | 58 | 62 |
| *ATG9A* | 57 | 65 |
| *ATG9B* | 49 | 52 |
| *ATP6V0C* | 54 | 59 |
| *ATP6V0D1* | 58 | 66 |
| *ATP6V0E1* | 55 | 58 |
| *ATP6V1A* | 53 | 58 |
| *ATP6V1B2* | 50 | 56 |
| *ATP6V1C1* | 56 | 61 |
| *ATP6V1D* | 56 | 63 |
| *ATP6V1E1* | 58 | 63 |
| *ATP6V1G1* | 55 | 61 |
| *ATP6V1G2* | 57 | 61 |
| *ATP6V1H* | 56 | 59 |
| *BCL2* | 58 | 62 |
| *BECN1* | 58 | 66 |
| *BNIP1* | 56 | 61 |
| *CAPN1* | 55 | 59 |
| *CAPN10* | 52 | 55 |
| *CAPNS1* | 58 | 62 |
| *CAPS* | 55 | 62 |
| *CDC37* | 57 | 64 |
| *CDK5* | 59 | 66 |
| *CDK5R1* | 54 | 60 |
| *CHMP2A* | 40 | 47 |
| *CHMP2B* | 43 | 47 |
| *CHMP4B* | 54 | 59 |
| *CISD2* | 46 | 49 |
| *CLEC16A* | 57 | 62 |
| *CRNKL1* | 52 | 59 |
| *CSNK2A1* | 59 | 62 |
| *CSNK2A2* | 54 | 61 |
| *CTSA* | 55 | 61 |
| *CTSB* | 57 | 63 |
| *CTSK* | 59 | 66 |
| *CTTN* | 58 | 61 |
| *DAP* | 55 | 55 |
| *DAPK3* | 53 | 59 |
| *DEPDC5* | 52 | 55 |
| *DNM1L* | 56 | 62 |
| *DPF3* | 56 | 60 |
| *DYNLL1* | 54 | 60 |
| *DYNLL2* | 57 | 64 |
| *EEF1A1* | 46 | 50 |
| *EI24* | 44 | 50 |
| *EMC6* | 55 | 56 |
| *EP300* | 48 | 51 |
| *EPM2A* | 57 | 60 |
| *EXOC1* | 38 | 42 |
| *EXOC4* | 51 | 54 |
| *EXOC7* | 53 | 59 |
| *FAM131B* | 58 | 64 |
| *FAM134B* | 57 | 61 |
| *FAM13B* | 57 | 62 |
| *FBXL2* | 57 | 61 |
| *FBXO7* | 57 | 62 |
| *FBXW7* | 56 | 62 |
| *FEZ1* | 38 | 43 |
| *FGF14* | 54 | 54 |
| *FGF7* | 56 | 61 |
| *FIS1* | 58 | 65 |
| *FLCN* | 56 | 61 |
| *FOXO1* | 57 | 62 |
| *FUNDC1* | 53 | 59 |
| *FUNDC2* | 54 | 59 |
| *GAPDH* | 50 | 55 |
| *GBA* | 54 | 60 |
| *GFAP* | 57 | 63 |
| *GOLGA2* | 48 | 54 |
| *GSK3A* | 51 | 57 |
| *HAPLN1* | 47 | 54 |
| *HGF* | 56 | 61 |
| *HIF1A* | 55 | 62 |
| *HK2* | 58 | 65 |
| *HMGB1* | 50 | 55 |
| *HSF2BP* | 54 | 56 |
| *HSP90AA1* | 47 | 52 |
| *HSPA8* | 53 | 58 |
| *HSPB1* | 55 | 57 |
| *HTR2B* | 56 | 63 |
| *HTRA2* | 56 | 62 |
| *HTT* | 56 | 58 |
| *IPPK* | 57 | 59 |
| *IST1* | 54 | 61 |
| *KDM4A* | 54 | 60 |
| *KIAA1549L* | 42 | 46 |
| *LAMP1* | 44 | 47 |
| *LAMTOR1* | 59 | 66 |
| *LAMTOR2* | 58 | 66 |
| *LAMTOR3* | 59 | 65 |
| *LAMTOR4* | 58 | 63 |
| *LAMTOR5* | 55 | 58 |
| *LARP1* | 55 | 62 |
| *LARP1B* | 55 | 59 |
| *LEP* | 58 | 65 |
| *LGALS8* | 58 | 63 |
| *LIX1* | 58 | 63 |
| *LIX1L* | 59 | 64 |
| *LSM4* | 41 | 43 |
| *LZTS1* | 37 | 41 |
| *MAP1LC3B* | 59 | 65 |
| *MAPK3* | 61 | 65 |
| *MAP3K7* | 55 | 62 |
| *MAPK8* | 53 | 60 |
| *MBD5* | 48 | 51 |
| *MCOLN1* | 57 | 64 |
| *MDH1* | 55 | 59 |
| *MET* | 55 | 59 |
| *MEX3C* | 48 | 55 |
| *MFN1* | 57 | 63 |
| *MFN2* | 57 | 61 |
| *MTCL1* | 45 | 48 |
| *MTM1* | 57 | 59 |
| *MTMR14* | 55 | 60 |
| *MTOR* | 47 | 47 |
| *MVB12A* | 53 | 58 |
| *MYOM1* | 57 | 59 |
| *NBR1* | 57 | 63 |
| *NEDD4* | 55 | 59 |
| *NPC1* | 59 | 64 |
| *NPRL2* | 39 | 45 |
| *NRBF2* | 54 | 60 |
| *NUP93* | 59 | 64 |
| *P2RX5* | 57 | 62 |
| *PARK7* | 57 | 58 |
| *PDK4* | 49 | 54 |
| *PEX13* | 56 | 62 |
| *PEX3* | 59 | 65 |
| *PHF23* | 56 | 62 |
| *PHYHIP* | 53 | 57 |
| *PI4K2A* | 55 | 58 |
| *PIK3R4* | 43 | 47 |
| *PIM2* | 48 | 51 |
| *PLK2* | 57 | 63 |
| *POLDIP2* | 38 | 42 |
| *POLR3A* | 39 | 43 |
| *PRKAB1* | 53 | 59 |
| *PRKAB2* | 40 | 44 |
| *PRKAG3* | 37 | 39 |
| *PRKD2* | 53 | 57 |
| *PRKG1* | 53 | 55 |
| *PSAP* | 60 | 66 |
| *PTK2* | 59 | 60 |
| *RAB12* | 57 | 58 |
| *RAB1A* | 59 | 65 |
| *RAB23* | 59 | 62 |
| *RAB24* | 59 | 63 |
| *RAB3GAP1* | 57 | 63 |
| *RAB33A* | 57 | 62 |
| *RAB33B* | 52 | 54 |
| *RAB39A* | 59 | 61 |
| *RAB5A* | 59 | 65 |
| *RAB7A* | 53 | 59 |
| *RALB* | 58 | 60 |
| *RASIP1* | 56 | 60 |
| *RB1CC1* | 55 | 59 |
| *RBM18* | 59 | 65 |
| *RGS19* | 57 | 60 |
| *RHEB* | 60 | 65 |
| *RNF152* | 55 | 60 |
| *RNF185* | 59 | 62 |
| *RNF41* | 58 | 65 |
| *RNF5* | 57 | 59 |
| *ROCK1* | 55 | 57 |
| *RPL28* | 57 | 61 |
| *RPS27A* | 58 | 59 |
| *RRAGA* | 57 | 64 |
| *RRAGC* | 57 | 64 |
| *RRAGD* | 59 | 64 |
| *RUVBL1* | 57 | 64 |
| *SCOC* | 58 | 62 |
| *SESN2* | 59 | 66 |
| *SFRP4* | 56 | 61 |
| *SH3BP4* | 57 | 62 |
| *SLC1A3* | 58 | 63 |
| *SLC22A3* | 53 | 56 |
| *SLC35C1* | 58 | 63 |
| *SMURF1* | 59 | 62 |
| *SNAPIN* | 60 | 64 |
| *SNCA* | 51 | 55 |
| *SNF8* | 55 | 61 |
| *SNRPB2* | 51 | 57 |
| *SNRPD1* | 40 | 45 |
| *SNRPF* | 57 | 62 |
| *SNTG1* | 53 | 54 |
| *SNX14* | 58 | 61 |
| *SNX5* | 59 | 65 |
| *SNX6* | 51 | 54 |
| *SOGA1* | 60 | 64 |
| *SPTLC1* | 55 | 56 |
| *SPTLC2* | 55 | 58 |
| *SQSTM1* | 59 | 63 |
| *SREBF2* | 42 | 48 |
| *SRPX* | 58 | 61 |
| *STOM* | 46 | 51 |
| *STX12* | 58 | 64 |
| *STX17* | 60 | 63 |
| *SUPT20H* | 55 | 59 |
| *SUPT5H* | 57 | 61 |
| *SVIP* | 46 | 46 |
| *TBC1D12* | 56 | 59 |
| *TBC1D14* | 55 | 57 |
| *TBC1D17* | 53 | 57 |
| *TBC1D25* | 57 | 61 |
| *TBC1D5* | 53 | 55 |
| *TBK1* | 58 | 63 |
| *TFEB* | 58 | 61 |
| *TIGAR* | 35 | 37 |
| *TM9SF1* | 54 | 60 |
| *TMBIM6* | 59 | 64 |
| *TMEM203* | 55 | 58 |
| *TMEM208* | 60 | 67 |
| *TMEM39A* | 60 | 64 |
| *TMEM39B* | 56 | 62 |
| *TMEM59* | 60 | 64 |
| *TNFAIP3* | 39 | 44 |
| *TOLLIP* | 57 | 60 |
| *TPCN1* | 54 | 59 |
| *TRAPPC8* | 60 | 62 |
| *TRIM13* | 57 | 64 |
| *TRIM8* | 52 | 57 |
| *TSC2* | 58 | 64 |
| *TSG101* | 40 | 45 |
| *TSPO* | 58 | 61 |
| *TXLNA* | 57 | 63 |
| *U2AF1* | 50 | 55 |
| *UBA52* | 55 | 55 |
| *UBB* | 43 | 44 |
| *UBC* | 37 | 37 |
| *UBQLN1* | 59 | 64 |
| *UBQLN2* | 54 | 58 |
| *UBQLN4* | 58 | 63 |
| *UBXN2B* | 59 | 60 |
| *ULK2* | 49 | 51 |
| *ULK3* | 55 | 62 |
| *USP13* | 43 | 46 |
| *UVRAG* | 44 | 45 |
| *VAMP8* | 52 | 58 |
| *VCP* | 50 | 55 |
| *VMP1* | 53 | 55 |
| *VPS25* | 53 | 58 |
| *VPS26B* | 54 | 58 |
| *VPS33A* | 51 | 55 |
| *VPS35* | 44 | 47 |
| *VPS37C* | 42 | 43 |
| *VPS37D* | 51 | 55 |
| *VPS39* | 59 | 64 |
| *VPS4A* | 40 | 44 |
| *WBP11* | 56 | 63 |
| *WDR24* | 53 | 59 |
| *WDR41* | 61 | 62 |
| *YIPF1* | 59 | 64 |
| *ZC3H12A* | 57 | 62 |
| *ZCCHC17* | 58 | 62 |
| *ZDHHC8* | 49 | 52 |
| *ZNF189* | 57 | 64 |
| *ZNF593* | 57 | 62 |
