# Supplementary material for: Evolution of mammalian longevity: age-related increase in autophagy in bats compared to other mammals
Source: Aging (Albany NY). 2021 Mar 21;13(6):7998–8025. doi: 10.18632/aging.202852 (PMC8034928; doi:10.18632/aging.202852)
Supplement: Supplementary Table 11 [file aging-13-202852-s011.pdf]

Supplementary Table 11. Additional gene data.

Mammalian genome	Number of genes	Order	Reference used
Rhinolophus ferrumequinum	160	Chiroptera	*Rhinolophus sinicus*
Megaderma lyra	100	Chiroptera	*Rhinolophus sinicus*
Eidolon helvum	174	Chiroptera	*Myotis lucifugus*
Pteronotus parnellii	108	Chiroptera	*Myotis lucifugus*
Myotis myotis	254	Chiroptera	*Myotis lucifugus*
Choloepus hoffmanni	168	Xenarthra	*Dasypus novemcinctus*
Procavia capensis	185	Afrotheria	*Loxodonta africana*
Manis pentadactyla	87	Pholidota	*Manis javanica*

A number of additional genomes were mined for autophagy-related genes. These data were included in a separate data set. The reference genome used to annotate each target mammal is displayed.
